# Spin‐Selective Anti‐Perovskite Enables Breakthrough Nitrate‐to‐Ammonia Electrocatalysis

**DOI:** 10.1002/adma.202523066

**Published:** 2026-02-12

**Authors:** Chun‐Kuo Peng, Hsiang‐Chun Yu, Shih‐Ching Huang, Yu‐Ru Lin, Suh‐Ciuan Lim, Jiayi Tang, Daqin Guan, Xiaomin Xu, Yijun Zhong, Yu‐Chang Lin, Zongping Shao, Yan‐Gu Lin

**Affiliations:** ^1^ Scientific Research Division National Synchrotron Radiation Research Center Hsinchu Taiwan; ^2^ Curtin Centre for Advanced Energy Materials and Technologies (CAEMT) Western Australian School of Mines (WASM) Curtin University Perth WA Australia; ^3^ Department of Materials Science and Engineering National Yang Ming Chiao Tung University Hsinchu Taiwan

**Keywords:** ammonia synthesis, antiperovskite, nitrate reduction reaction, operando X‐ray absorption spectroscopy, operando X‐ray emission spectroscopy, spin‐selective catalysis

## Abstract

Electrochemical nitrate reduction to ammonia offers environmental and energy benefits, but progress is hindered by sluggish multistep proton‐coupled electron transfers and competing side reactions. Here, we introduce an antiperovskite CuNCo_3_ catalyst featuring a 3*d*–3*d* interaction framework. This framework stabilizes spin‐selective Co sites even upon surface Co‐N bond cleavage and drives asymmetric nitrate consumption. CuNCo_3_ achieves 100% Faradaic efficiency and an NH_3_ production rate of 124.6 mg mg_cat_^−1^ h^−1^ at −0.4 V vs. RHE. *Operando* XAS, XES, and ATR‐FTIR directly link the evolution of spin‐selective Co sites with specific NO_3_RR intermediates, revealing that spin‐selective Co sites lower hydrogenation barriers and accelerate key steps. These results demonstrate that spin‐selective anti‐perovskite frameworks provide a robust, earth‐abundant platform for high‐performance nitrate‐to‐ammonia electrocatalysts.

## Graphical abstract

CuNCo_3_ antiperovskite leverages built‐in 3*d*–3*d* interactions to stabilize and generate spin‐selective Co sites during nitrate reduction. Multiple *operando* techniques reveal that Cu acts as a promoter, inducing partial surface Co‐N cleavage and Cu‐Co charge redistribution. This dynamic restructuring tunes the activation and conversion of nitrate‐derived intermediates, boosting overall catalytic efficiency and selectivity for ammonia formation.

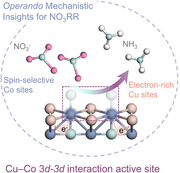

## 1 Introduction

Probing the reaction mechanism of nitrate reduction to ammonia is key to improving both rate and selectivity, and it also guides the development of highly active and durable electrocatalysts. Nitrate reduction reaction (NO_3_RR) involves multistep proton‐coupled electron transfers and multiple reaction branches, and it exhibits complex and sluggish kinetics [1, 2]. Its rate and selectivity are mainly limited by the first deoxygenation‐hydrogenation and the first hydrogenation steps [2, 3, 4]. To date, precisely controlling and quantitatively elucidating catalyst spin states to accelerate *NO hydrogenation remains challenging. Given that NH_3_ serves both as a fertilizer precursor and a carbon‐free energy carrier [5, 6], the selective electroreduction of nitrate to ammonia offers a viable route to address energy and environmental pressures, which makes clarifying the key NO_3_RR mechanism especially important [7].

Tuning the electronic structure of metal sites is pivotal for simultaneously optimizing the adsorption of all NO_3_RR intermediates [8, 9]. In particular, spin polarization offers a fast spin‐exchange channel for the critical de‐oxygenation and hydrogenation steps [10, 11, 12]. Owing to adjustable crystal‐field splitting, transition metals possess rich spin freedom that has proven decisive in oxygen evolution reaction, oxygen reduction reaction, and related reactions [13, 14]. For NO_3_RR, spin sites accelerate NO_3_^−^ consumption, favor asymmetric adsorption configurations, lower subsequent hydrogenation barriers, and ultimately enhance NH_3_ selectivity. Yet correlating the *operando* evolution of metal‐site spin states with specific intermediates remains challenging.

Cu‐based catalysts suppress the hydrogen evolution reaction (HER), and their relatively high d‐electron density promotes NO_3_^−^ adsorption, leading to widespread use for nitrate reduction [4, 15]. However, under operating potentials, their structures and electronic states often undergo dynamic evolution. A key issue is the accumulation of NO_2_^−^, which both accelerates catalyst deactivation and renders the subsequent hydrogenation steps toward NH_3_ kinetically sluggish [16]. In addition, at low NO_3_^−^ concentrations or at more negative potentials, competition from HER becomes pronounced, making it difficult to maintain high NH_3_ selectivity over extended operation [17, 18, 19]. Co‐based catalysts are also promising candidates [20, 21], but their active species and reaction mechanism remain unclear. Many studies indicate that their surface and electronic structures are modulated by the applied potential, thereby shifting the reaction pathway [22, 23]. Consequently, suppressing HER and strengthening rate control over the key hydrogenation and deoxygenation steps still require further design and validation [24, 25]. Taken together, there is an urgent need to develop metal frameworks that simultaneously stabilize key active sites and cooperatively regulate the adsorption of critical intermediates, so as to balance reaction rate with NH_3_ selectivity.

Here, we introduce an anti‐perovskite 3*d*–3*d* framework in which a linear Co‐N‐Co channel, coupled with a Cu‐site *s*/*p* level, builds a tunable 3*d*–3*d* interaction coupling. This 3*d*–3*d* interaction stabilizes the spin electron of Co sites while continuously modulating intermediate adsorption and conversion, achieving a concerted enhancement of rate and selectivity. The framework tolerates surface Co‐N bond cleavage and generates distinctive Co‐selective sites that drive asymmetric nitrate consumption, delivering high NH_3_ yield and selectivity. Using *operando* X‐ray absorption spectroscopy (XAS), X‐ray emission spectroscopy (XES), and attenuated total reflection‐Fourier‐transform infrared absorption spectroscopy (ATR‐FTIR), we dynamically track the evolution of electronic structure, spin state, and intermediates, directly confirming the formation and stability of spin‐selective Co sites and clarifying their intrinsic role in boosting NO_3_RR activity. These findings establish the anti‐perovskite 3*d*–3*d* framework as a viable platform for designing efficient, stable, and spin‐regulated nitrate‐to‐ammonia electrocatalysts.

## 2 Results and Discussion

### 2.1 Catalyst Characterization

**FIGURE 1:**
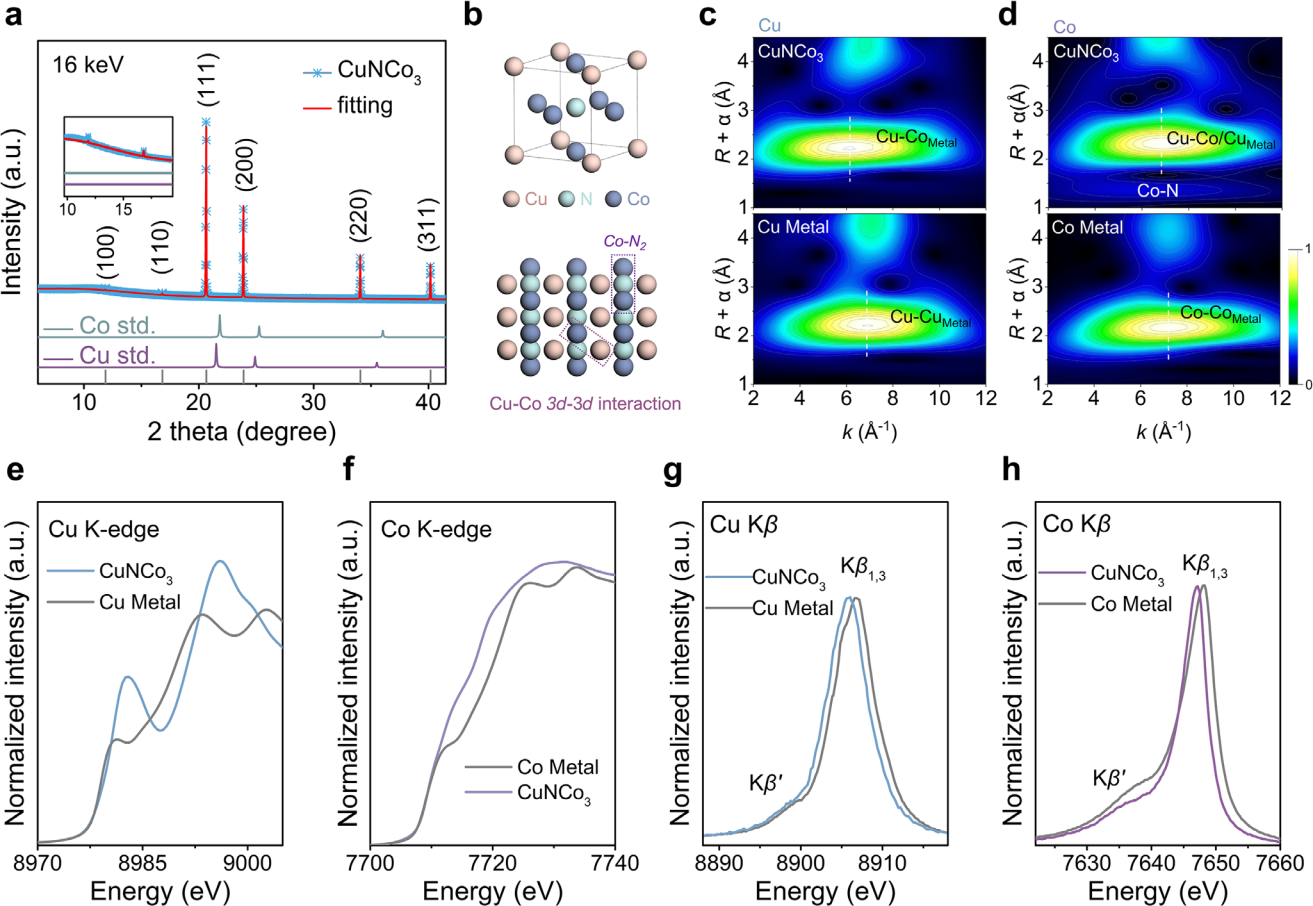
Atomic and electronic structure characterization of CuNCo_3_ catalysts. (a) Rietveld refined XRD pattern of CuNCo_3_. (b) Crystal structure of the CuNCo_3_ antiperovskite. WT‐EXAFS contour plots for the (c) Cu and (d) Co K‐edge in CuNCo_3_ alongside the corresponding metal foils. (e) Cu and (f) Co K‐edge XANES spectra of CuNCo_3_ compared with metallic references. (g) Cu and (h) Co K*β* XES spectra of CuNCo_3_ and references.

The X‐ray diffraction (XRD) pattern of the CuNCo_3_ anti‐perovskite shows a peak shift compared to metallic peaks, and a characteristic peak appears at a low angle. The XRD refinement results further indicate the anti‐ReO_3_ crystal structure in space group Pm‐3m, as shown in Figure 1a and Table S1, while the corresponding crystal model is illustrated in Figure 1b. The scanning electron microscope (SEM) images reveal a cube‐like morphology (Figure S1). As shown in Figure S2a, high‐resolution transmission electron microscope (HR‐TEM) images and the corresponding fast Fourier transform (FFT) patterns further confirm the successful synthesis of CuNCo_3_. Energy‐dispersive X‐ray spectroscopy (EDS) in Figure S2b shows a uniform distribution of elements with a Co:Cu weight ratio of 1:3 (as shown in Figure S3). Inductively coupled plasma mass spectrometry (ICP) also shows consistent results (Table S2). The *k* ^2^χ(*k*) oscillation functions for Co and Cu are distinct from those of their respective metal foil (Figure S4). The wavelet transform‐extended X‐ray absorption fine structure (WT‐EXAFS) of Cu and Co (Figure 1c,d) shows a central feature at 6 Å^−1^ that is negatively shifted compared to their metal standards, corresponding to the scattering paths of Cu─Co and Co─Co/Cu, respectively. Moreover, a weak Co‐N peak was observed in the WT‐EXAFS of Co (Figure 1d). Subsequently, the fitted EXAFS spectra for Co and Cu, presented in Figure S5 and Table S3, respectively, provide a clearer identification of the metal‐metal coordination environment of 3*d*–3*d* interaction in CuNCo_3_. Notably, Co exhibits both Co─Co/Cu and Co─N covalency. To gain deeper insight into the unique 3*d*–3*d* interaction in CuNCo_3_, Cu and Co K‑edge X‐ray absorption near‐edge structure (XANES) were conducted to analyze the electronic structure. Figure 1e shows that the Cu K‐edge white‐line intensity is markedly higher than that of metallic Cu. This enhancement indicates an increased 4*p* hole concentration, which results from strong hybridization between the bonding Cu 4*s*/4*p* orbitals and the Co 3*d* orbitals [26, 27]. As shown in Figure 1f, the Co K‑edge spectra occur at an energy position similar to that of metallic Co, but a shoulder appears at around 7714 eV, corresponding to Co─N coordination [28] and consistent with previous EXAFS results. First‑derivative spectra of both Cu and Co also reveal clear valence shifts (Figure S6). These findings indicate that the apparent 3*d*–3*d* interaction is actually mediated through Cu 4*s*/4*p*‐N 2*p*‐Co 3*d* hybridization [26, 27]. The high‐resolution X‐ray photoelectron spectroscopy (XPS) results for Co, Cu, and N also show consistent findings (Figure S7). Combining the Cu XPS and Auger spectra, it can be confirmed that Cu is in the Cu^+1^ state(Figure S7a,b). XPS of Co 2*p*_3/2_ in Figure S7c,d observed partial valence states, indicating cobalt bond with nitrogen. The magnetic hysteresis loop at 300 K in Figure S8 shows unexpectedly lower magnetic behavior in CuNCo_3_ compared to metallic Co, suggesting the presence of fewer unpaired electrons. This can also be well supported by XES of Co and Cu Figure 1g,h), where the intensity of the Co K*β*′ peak is lower in the spectra compared to the Co metal reference, indicating a lower spin state. Meanwhile, the K*β*_1,3_ main peaks of both Cu and Co shift to higher energies relative to their respective metal references, indicating that both elements are present in valent states (Figure 1g). As for Cu, since its electrons are fully filled, exhibiting no spin state, only a shift in the K*β*_1,3_ peak (Figure 1h). This configuration of Cu, utilizing the 4*s* and 4*p* orbitals along with the unique valence state of Co sites, could exhibit higher catalytic activity and stability for NO_3_^−^ reduction reaction.

### 2.2 Electrochemical Properties Toward NO_3_^−^ Reduction Activity

**FIGURE 2:**
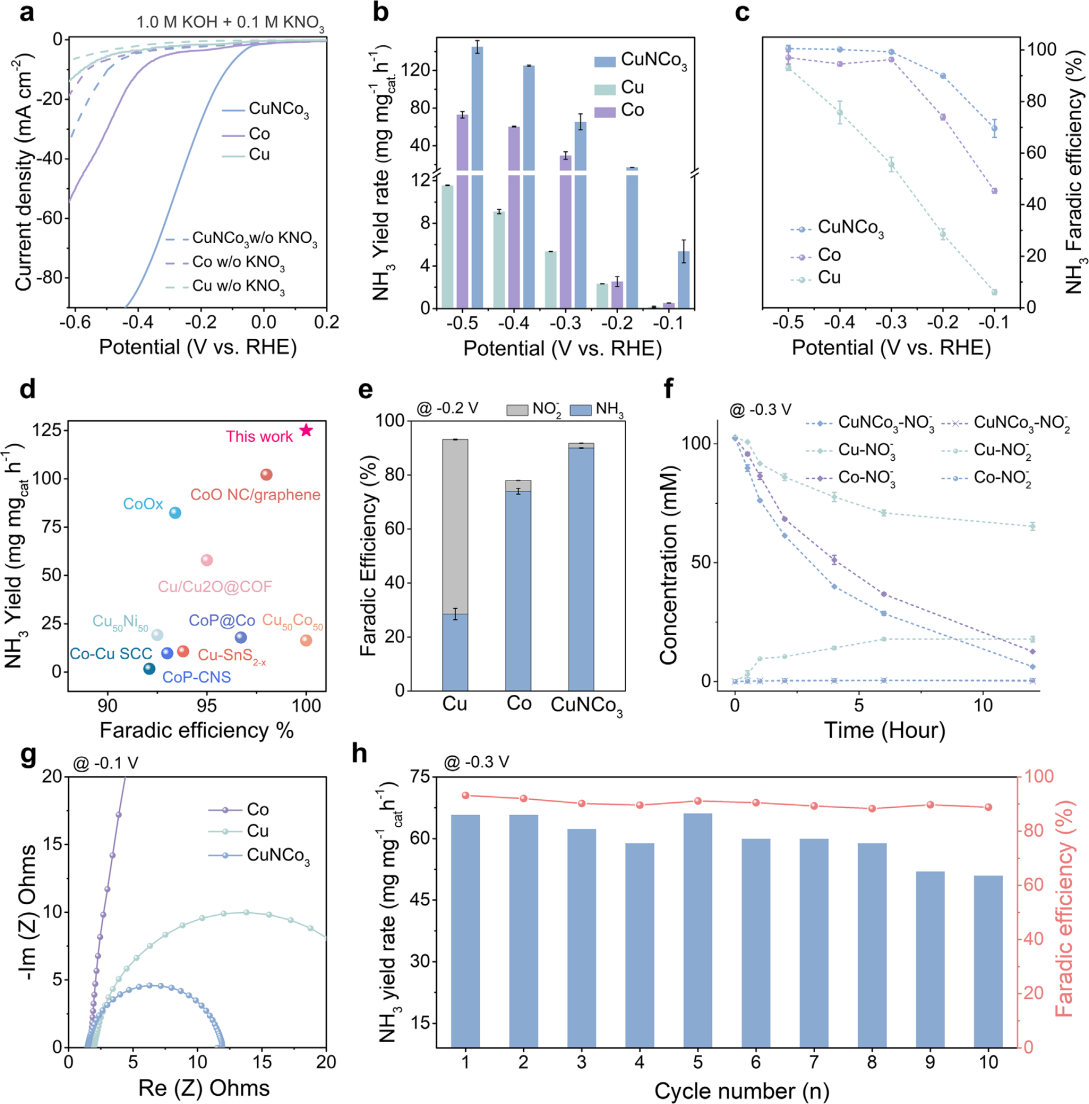
NO_3_RR performance of anti‐perovskite CuNCo_3_ catalyst. (a) LSV curves of the different catalysts recorded in 1.0 M KOH with 0.1 M KNO_3_ (solid lines) and in nitrate‐free 1.0 M KOH (dashed lines). (b) Ammonia yield rate as a function of applied potential. (c) Faradaic efficiency comparison of different samples. (d) Nitrate reduction reaction performance compared with the reported literature. (e) Comparison of nitrite byproduct formation at −0.3 V vs. RHE. (f) Kinetic analysis of nitrate consumption behavior for CuNCo_3_ and metal catalysts. (g) Nyquist plots recorded at −0.1 V vs. RHE for CuNCo_3_ and metal catalysts. (h) Long‐term stability test of CuNCo_3_ at −0.3 V vs. RHE.

Figure 2a shows *iR*‐corrected Linear sweep voltammetry (LSV) for each catalyst in 1.0 M KOH with 0.1 M KNO_3_. Compared with pure Co and Cu, CuNCo_3_ exhibits a positive shift of 0.1 V vs. RHE in onset potential and higher current density. All samples were then underwent to 0.5 h electrolysis at different potentials (Figure S9a–c). Figure 2b shows that CuNCo_3_ exhibits the highest ammonia yield rate of 124.6 mg mg_cat_^−1^ h^−1^ at −0.4 V vs. RHE, nearly twice that of the Co metal. The Faradaic efficiency values for each individual catalyst are provided in Figure S9d–f. Cu catalyst exhibits persistently low Faradaic efficiency toward NH_3_, with noticeable NO_3_^−^ formation. In contrast, for Co catalyst, the Faradaic efficiency increases with more negative potentials, but the rise remains comparatively slower than on CuNCo_3_ (Figure 2c). Notably, CuNCo_3_ achieved a higher Faradaic efficiency of 100% for ammonia at −0.3 V vs. RHE. These results indicate that the 3*d*–3*d* interaction framework is critical for NO_3_RR. Compared with metallic Co with unpaired spins (Figure 1h), CuNCo_3_ delivers higher ammonia yield and Faradaic efficiency and remains highly competitive against other alloyed catalysts (Figure 2d). This advantage stems from its distinctive electronic structure and higher electronic conductivity, which synergistically promote electron transfer and intermediate conversion, thereby accelerating the overall cascade kinetics. Accordingly, an adaptive 3*d*–3*d* interaction framework represents an effective design strategy for high‐performance NO_3_RR. The NH_3_ and NO_2_^−^ were quantified by UV–vis indophenol and Griess assays, respectively (Figures S10 and S11). To evaluate the NO_3_RR performance at varying nitrate concentrations and to examine the competing HER, we conducted NO_3_RR experiments using CuNCo_3_ at nitrate concentrations of 1, 10, and 100 mM (see Figure S21). The results show a clear dependence of the current density on nitrate concentration. Specifically, increasing the concentration to 100 mM led to a significant enhancement in the NO_3_RR LSV current, along with a notable increase in ammonia FE, reaching nearly 90% (Figure S21a,b). The corresponding NH_3_ yield rate is provided in Figure S21c. These findings indicate that CuNCo_3_ exhibits higher NO_3_RR activity and selectivity at elevated nitrate concentrations. Evidently, HER competition becomes more prominent at low nitrate concentrations, posing a major challenge for selective NO_3_RR. To verify the origin of the detected NH_3_ and calibrate the ammonia yield, we performed ^1^H NMR measurements using an electrolyte that contained 0.1 M ^15^N‐labelled ^15^NO_3_^−^ (Figure S12). Control experiments conducted in pure KOH confirmed that no NO_x_ impurities interfered with the measurements (Figure S13). These results further demonstrate that nitrogen from the CuNCo_3_ lattice is not involved in the reaction. Figure 2d demonstrates the competitiveness of our study by comparing the ammonia yield rate and Faradaic efficiency of CuNCo_3_ with other reported NO_3_RR electrocatalysts [22, 29, 30, 31, 32, 33, 34, 35, 36]. Figure 2e further shows that CuNCo_3_ maintains higher NH_3_ selectivity than the metal references at lower potentials. Only Cu exhibits clear NO_2_^−^ formation. Kinetic analysis (Figure 2f) shows that CuNCo_3_ consumes NO_3_^−^ most rapidly at −0.3 V. Within 12 h, nearly all of the 100 mM NO_3_^−^ is depleted with no detectable NO_2_^−^, indicating that its unique Cu‐Co electronic structure suppresses accumulation of the nitrite intermediate. Nitrate conversion was calculated using a UV‐based calibration curve (Figure S14). To exclude the influence of different electrochemical surface area (ECSA) on NO_3_RR performance, the ECSA of each catalyst was determined (Figure S15). CuNCo_3_ has a larger surface area than the metallic samples. After normalization by ECSA (Figure S16), CuNCo_3_ still delivers the highest current, confirming excellent intrinsic electrocatalytic activity. Figure 2g further shows that CuNCo_3_ exhibits the lowest charge‐transfer resistance in the electrochemical impedance spectra (EIS). These observations demonstrate that the unique 3*d*–3*d* interaction framework delivers high intrinsic activity toward NO_3_RR, enabling efficient conversion. To more directly examine the intrinsic capability of the catalyst for converting key *NO intermediates, NO_2_RR control experiments were performed. Cu, Co, and CuNCo_3_ were evaluated in 0.1 M KNO_2_ + 1 M KOH, where CuNCo_3_ still delivers the best overall catalytic activity (Figure S22a). Notably, relative to NO_3_RR, Cu shows a lower LSV current in NO_2_RR, accompanied by decreased NH_3_ FE and yield rate (Figure S22b,c). This trend is consistent with Cu being more effective in promoting the initial NO_3_^−^ to NO_2_^−^ conversion [37], whereas NO_2_RR relies more on the subsequent hydrogenation and deoxygenation steps, making Cu comparatively less favorable under these conditions. In contrast, Co shows a more pronounced activation in NO_2_RR, with a much larger increase in NH_3_ yield rate (Figure S22c), suggesting that the NO_3_^−^ to NO_2_^−^ conversion step plays a more critical role in governing the overall rate on Co. By comparison, CuNCo_3_ also becomes activated in NO_2_RR, but the change is less evident, indicating reaction characteristics distinct from metallic Cu and Co. Figure S22d further shows that the NH_3_ yield rate increases by ∼300% for Co in NO_2_RR, whereas the increase is ∼46% for CuNCo_3_, collectively indicating that the outstanding NO_3_RR performance of CuNCo_3_ is more closely related to its intrinsic structural characteristics. As shown in Figure 2h, the Faradaic efficiency remained stable during a 10‐cycle continuous electrolysis test at −0.3 V vs. RHE. As shown in Figure S23a, the current density remains stable over 24 h, and both the ammonia yield rate and Faradaic efficiency exhibit no significant decline, confirming the robust operational stability of the catalyst (Figure S23b). Furthermore, to assess potential structural degradation after prolonged electrolysis, we performed post‐reaction XRD and SEM analyses. As shown in Figure S24a,b, the lattice structure remains well preserved following the stability test. ICP analysis shows negligible Cu and Co dissolution from CuNCo_3_, with both metal concentrations remaining below 10 ppb (Table S7). The results indicate that the lattice structure remains well preserved after the stability test, demonstrating the electrochemical stability and reliability of the CuNCo_3_ catalyst.

### 2.3 Ligand‐Interaction Reaction Mechanism

**FIGURE 3:**
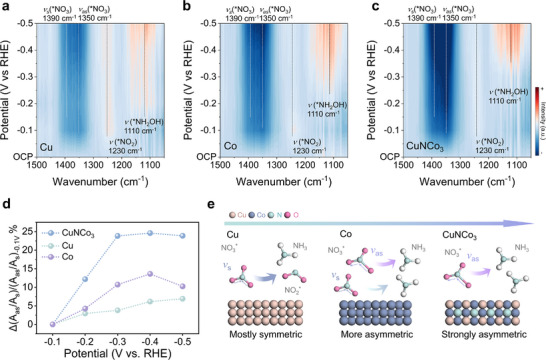
*Operando* ATR‐FTIR mechanistic study of NO_3_RR. *Operando* ATR‐FTIR spectra of (a) CuNCo_3_, (b) Cu, and (c) Co with potential scan from OCP to −0.5 V vs. RHE. (d) Trend of symmetric and asymmetric peak ratio. (e) Proposed ligand‐interaction reaction mechanism for nitrate reduction.

*Operando* ATR‐FTIR experiments clearly captured the dynamic evolution of surface nitrate species and further clarified the ammonia‐formation mechanism. The 2D contour plots in Figure 3a–c display four key absorption bands at 1110, 1230, 1350, and 1390 cm^−1^. Negative signals at 1350 and 1390 cm^−1^ which were assigned to the asymmetric *v*_as_ (N─O) and symmetric *v*_s_ (N─O) modes respectively, reflect the consumption of the corresponding NO_3_^−^ ligand vibration. The NO_3_^−^ is planar with D_3_h symmetry, and its excess negative charge is delocalized over the entire molecule, making the three N─O bonds equivalent under ideal conditions. The so‐called asymmetric adsorption can be attributed to a breaking of the local symmetry by the surrounding environment. When hydrogen bonding or interfacial interactions preferentially involve one N─O bond, this local asymmetry can lead to transient elongation of that N─O bond and charge redistribution within the NO_3_^−^ ion [38, 39]. Such asymmetric adsorption can render the subsequent protonation/hydrogenation steps more favorable and potentially accelerate the reaction kinetics, thereby helping to enhance NO_3_RR performance. Positive peaks signify the accumulation of new species, with 1230 cm^−1^ assigned to NO_2_^−^ and 1110 cm^−1^ to NH_2_OH [40, 41]. Figure 3a shows that after applying potential, the blue‐shaded integral regions at 1350 and 1390 cm^−1^ for *v*_as_ and *v*_s_ on Cu do not darken appreciably, indicating the slowest nitrate consumption. In contrast, on Co, these blue‐shaded areas deepen markedly, indicating a higher fraction of asymmetric nitrate than on Cu (Figure 3b). Figure 3c further shows that on CuNCo_3_ the *v*_as_ (N─O) bands grow much more strongly than on either single metal, demonstrating the highest nitrate‐consumption rate. The weaker consumption capability of Co toward *NO_3_ compared with CuNCo_3_ may be associated with the strong hydrogen affinity of Co. Recent studies have highlighted the crucial role of active hydrogen (*H) in NO_3_RR [42, 43]. Co often binds *H too strongly, such that under operating potentials *H may occupy a substantial fraction of active sites [34, 44]. This can suppress effective *NO_3_ adsorption and activation, limiting the achievable NO_3_RR Faradaic efficiency, consistent with the lower FE of Co (Figure 2c). By contrast, CuNCo_3_, enabled by its intrinsic structural characteristics and the associated 3*d*–3*d* interaction, can to some extent, tune the adsorption properties of the Cu‐Co sites. Such cooperative regulation may mitigate excessive *H occupation and provide more favorable surface conditions for *NO_3_ adsorption and subsequent conversion, thereby promoting improved NO_3_RR efficiency and selectivity. Notably, a nitrite peak at 1230 cm^−1^ appears only on metallic Cu, and it emerges already at −0.1 V. For Co and CuNCo_3_ the NO_2_^−^ signal remains negligible, consistent with the electrochemical observations above. To quantify these observations, we fitted the symmetric and asymmetric peaks (Figure S17 and Table S4) and summarized the trends in Figure 3d. The results show that an increased consumption ratio of asymmetric to symmetric N‐O vibrations is consistent with the preceding electrochemical trends, implying that preferential consumption of the asymmetric nitrate accelerates nitrate reduction. Notably, the *v*_as_/*v*_s_ ratio of CuNCo_3_ saturates at −0.3 V vs. RHE, consistent with earlier Faradaic efficiency showing CuNCo_3_ reaches 100% at −0.3 V. This indicates a close correspondence between the spectroscopic descriptor and Faradaic efficiency. In contrast, Co shows a decrease in asymmetric nitrate consumption at −0.5 V vs. RHE. Figure S18 shows that the partial current density of NH_3_ for CuNCo_3_ keeps rising from −0.4 to −0.5 V vs. RHE, and the increases for Co and Cu slow down. This trend of partial current density aligns with the *v*_as_/*v*_s_ ratio, exhibiting that the unique structure of CuNCo_3_ maintains an elevated *v*_as_/*v*_s_ ratio. A larger proportion of asymmetric nitrate corresponds to higher NH_3_ selectivity and yield. Mechanistically, in the asymmetric mode, compared with the symmetric mode, NO_3_^−^ is more prone to local symmetry breaking, which leads to an uneven distribution of bond lengths and charges and renders a specific oxygen terminus more reactive [38, 39]. Figure 3e indicates that asymmetric nitrate becomes progressively more prevalent on Cu, then Co, and is highest on CuNCo_3_. On Cu, nitrate remains largely in the symmetric configuration, limiting its electrochemical consumption rate and is consistent with the preceding electrochemical results. In contrast, on CuNCo_3_, the distinctive 3*d–3d* interaction framework promotes the formation of asymmetric nitrate intermediates and accelerates their conversion to ammonia.

### 2.4 Identification of the Active Site

**FIGURE 4:**
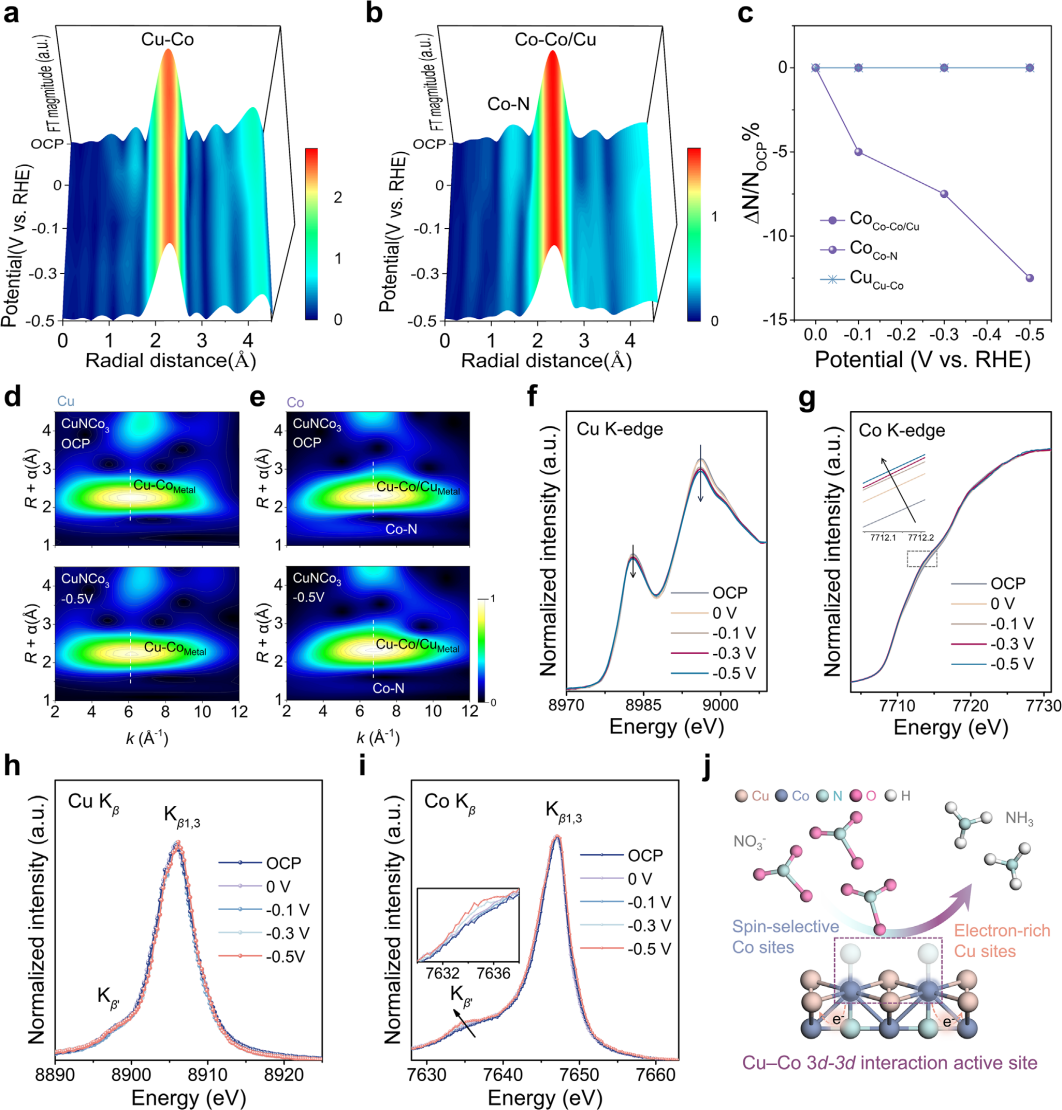
*Operando* quick‐XAS and XES characterizations for NO_3_RR. 3D patterns of *operando* (a) Cu and (b) Co K‐edge EXAFS of CuNCo_3_. (c) Fitting‐derived coordination number trends of CuNCo_3_. WT‐EXAFS spectra of (d) Cu and (e) Co comparison under *operando* applied potentials. *Operando* (f) Cu and (g) Co K‐edge XANES of CuNCo_3_. *Operando* XES spectra of (h) Cu and (i) Co of CuNCo_3_. (j) Proposed reaction mechanism for nitrate reduction on CuNCo_3_.

The electronic structures of the real active sites in CuNCo_3_ catalysts during the nitrate reduction reaction were well assessed using *operando* quick‐XAS and *operando* XES (Figure 4). The *operando* EXAFS spectra of Cu and Co K‐edge were firstly measured to probe the dynamic structural variation. In Figure 4a,b, the 3D Cu and Co EXAFS reveal essentially unchanged bond lengths, while only the Co─N coordination number shows a slight reduction. The detailed trend of coordination numbers for Co and Cu centers is summarized quantitatively in Figure 4c. Specifically, the Co─N peak intensity at −0.5 V vs. RHE vs. the open circuit potential (OCP) drops by 12.5%, implying partial exposure of bare Co sites for NO_3_RR. The corresponding *k* ^2^χ(*k*) oscillation functions and the fitted profiles of EXAFS spectra for Co and Cu are presented in Figures S19, S20, and Tables S5, S6, respectively. As shown in Figure 4d,e WT‐EXAFS spectra clearly investigated the local geometry of Cu and Co sites during the nitrate reduction reaction. WT‐EXAFS spectra of Cu in Figure 4d show a characteristic peak of Cu─Co at around 6.6 Å^−1^. The coordination environment of Cu remains unchanged during the NO_3_RR process. In Figure 4e, the Co─N signal at 5.6 Å^−1^ weakens under −0.5 V vs. RHE relative to OCP, consistent with the EXAFS findings. The *operando* Cu and Co K‐edge XANES spectra (Figure 4f,g) are consistent with the results described above. The Cu XANES spectra exhibit a slight increase in electron density, suggesting that charge transfer from the Co site helps maintain continuous catalysis (Figure 4f). Only the Co XANES spectra show slight shoulder shape changes, consistent with EXAFS results and suggesting partial Co─N bond cleavage and direct interaction of Co sites centers with nitrate. Owing to the unique Cu─Co interaction, the lattice remains intact despite partial Co─N bond cleavage, as charge redistribution between Cu and Co cooperatively stabilizes the framework. To further probe the spin states of the catalysts during NO_3_RR process, *operando* Cu and Co X‐ray emission spectroscopy were recorded. The XES measurements on the K*β* emissions of Co and Cu at the reduction potential (Figure 4h,i). The Cu K*β* spectra remain unchanged (Figure 4h), in line with the XAS results. Meanwhile, *operando* Co XES reveals that applying a negative potential increases the intensity of the K*β*′ peak, indicating a spin transition in Co that generates spin‐selective sites capable of participating in NO_3_^−^ reduction (Figure 4i). This phenomenon aligns with the *operando* ATR‐FTIR observation of stronger *v*_as_ (N─O) stretching bands and is linked to the formation of spin‑selective Co sites. It suggests that partial Co‐N bond cleavage, together with Cu‐Co charge redistribution, increases the Co spin state, thereby enhancing *v*_as_ (N─O) activation and accelerating NH_3_ production. Figure 4j schematically illustrates the electron redistribution in Co and Cu. The proposed mechanism involves charge transfer from Co to Cu accompanied by the formation of spin‐selective Co sites, collectively boosting NO_3_RR performance.

Complementary *operando* experiments reveal the atomic and electronic rearrangements that occur in CuNCo_3_ during nitrate reduction. A unique Cu─Co 3*d‐*‐3*d* interaction creates a highly active platform. *Operando* spectroscopy shows that within this 3*d*–3*d* framework, Co acts as the true active site, becoming spin‐selective under reaction conditions, while Cu mediates electron transfer. This cooperation allows nitrate to release partially negatively charged oxygen atoms, thereby accelerating ammonia formation.

## 3 Conclusions

As demonstrated by the CuNCo_3_ antiperovskite, a 3*d‐*‐3*d* interaction framework can elucidate and harness the critical role of spin‐selective sites in nitrate reduction. *Operando* XAS, XES, and ATR‐FTIR spectroscopies provide the first direct evidence of spin‐selective Co sites in the antiperovskite and correlate asymmetric nitrate adsorption with the evolution of the metal‐site electronic structure. Under operating potentials, partial cleavage of surface Co‐N bonds generates these spin‐selective centers, while Co to Cu charge redistribution preserves the integrity of the bulk lattice. The spin‐selective Co sites enhance asymmetric nitrate adsorption, accelerate proton‐coupled electron transfer, and achieve 100% Faradaic efficiency for ammonia production. This spin‐engineered electronic scaffold differs markedly from conventional alloys or simple nitrides, demonstrating that a homogeneous 3*d* framework with spin‐selective Co sites is essential for highly selective nitrate‐to‐ammonia catalysis.

## Author Contributions

C.K.P. and Y.G.L. conceived and designed the project. C.K.P. carried out sample preparation and analyzed the overall experimental data under the supervision of Y.G.L. and Z.S. H.C.Y., and S.C.H. were primarily responsible for electrochemical measurements, with D.G., J.T., X. X., and Y.Z. providing additional support. Y.C.L., Y.R.L., and S.C.L. supported the *operando* hard‐XAS experiments. C.K.P., H.C.Y., and S.C.H. analyzed all *operando* data. All other authors discussed the results and assisted in manuscript preparation. Y.G.L. was responsible for the project management.

## Conflicts of Interest

The authors declare no conflicts of interest.

## Supporting information




**Supporting File**: adma72549‐sup‐0001‐SuppMat.docx.

## Data Availability

All data are available from the corresponding author upon reasonable request.
