# Supplementary material for: Spin‐Selective Anti‐Perovskite Enables Breakthrough Nitrate‐to‐Ammonia Electrocatalysis
Source: Adv Mater. 2026 Feb 12;38(16):e23066. doi: 10.1002/adma.202523066 (PMC12994318; doi:10.1002/adma.202523066)
Supplement: Supplementary file 1 — Supporting File: adma72549‐sup‐0001‐SuppMat.docx. [file ADMA-38-e23066-s001.docx]

Supporting Information

Spin-Selective Anti-perovskite Enables Breakthrough Nitrate-to-Ammonia Electrocatalysis

*Chun-Kuo Peng ^A^, Hsiang-Chun Yu ^A^, Shih‐Ching Huang ^A^, Yu-Ru Lin ^A^, Suh-Ciuan Lim ^A^, Jiayi Tang ^B^, Daqin Guan ^B^, Xiaomin Xu ^B^, Yijun Zhong ^B^, Yu-Chang Lin ^A^ , Zongping Shao ^B*^, Yan-Gu Lin ^A,C*^*

A. Chun-Kuo Peng, Hsiang-Chun Yu, Shih‐Ching Huang, Yu-Ru Lin, Suh-Ciuan Lim, Yu-Chang Lin, Yan-Gu Lin
Scientific Research Division, National Synchrotron Radiation Research Center, Hsinchu 300092, Taiwan.

E-mail: [lin.yg@nsrrc.org.tw](mailto:huang.sc@nsrrc.org.tw；lin.yg@nsrrc.org.tw)

B. Jiayi Tang, Daqin Guan, Xiaomin Xu, Yijun Zhong, Zongping Shao
Curtin Centre for Advanced Energy Materials and Technologies (CAEMT), Western Australian School of Mines (WASM), Curtin University, Perth, WA 6102, Australia.

E-mail: [Zongping.Shao@curtin.edu.au](mailto:Zongping.Shao@curtin.edu.au)

C. Yan-Gu Lin

Department of Materials Science and Engineering, National Yang Ming Chiao Tung University, Hsinchu, 300093, Taiwan.

Experimental Section

**Chemicals and Reagents.** Cobalt nanopowder (Alfa Aesar, 99.8%), Copper nanopowder (Alfa Aesar, 99.9%). Potassium nitrate (KNO_3_, Thermo scientific, 99%). Potassium hydroxide (KOH, Sigma-Aldrich, ACS reagent, ≥85%). Nafion^TM^117 solution (Sigma-Aldrich, 5wt%). Salicylic acid (Thermo scientific, 99%). Sodium hypochlorite solution (Showa, active chlorine~12%). Sodium pentacyanonitrosylferrate (III) dihydrate (Alfa Aesar, 98+%). N-(1-naphthyl) ethylenediamine dihydrochloride (Thermo scientific, ACS reagent, 98+%). Sulfanilamide (Alfa Aesar, 98%). Trisodium citrate dihydrate (Showa, 99%). Sodium hydroxide (NaOH, Showa, 97%). Phosphoric acid (H_3_PO_4_, Sigma-Aldrich, 85 wt.%, 99.3%; ρ = 1.70 g mL^-1^). Sulfamic acid (Sigma-Aldrich, ACS reagent, 99.3%). Potassium nitrate-^15^N (Sigma-Aldrich, ≥98 atom% ^15^N). All the chemicals were used as purchased without further purification.

**Preparation of CuNCo_3_ catalyst.** Cu and Co metal powders mixed in a weight ratio of 1:3, with a total weight of 50 mg. After thoroughly mixing the two metal powders, transfer them to a tubular furnace. Next, evacuate the atmosphere inside the furnace to 10^-3^ torr, then introduce a sufficient amount of ammonia gas and maintain a continuous flow of ammonia at a rate of 100 standard cubic centimeters per minute. Subsequently, heat the furnace to 400 °C with a heating rate of 4.5 °C per minute, and hold at this temperature for 3 hours. After the sample cools to room temperature, remove it and press it into pellets. Then, place the sample back in the same atmosphere for a second heating. This time, heat to 500 °C with a heating rate of 5.5 °C per minute and hold at this temperature for 6 hours. Finally, allow the sample to cool to room temperature before removing it, successfully obtaining the perovskite CuNCo_3_.

**Characterization.** The morphology of the catalysts was characterized using SEM (JEOL, JSM-6700F) and TEM (JEOL, ARM-200FTH). The high-resolution powder XRD was performed at TPS BL-19A beamline of NSRRC in Taiwan. The high-resolution XPS were measured at TLS BL-24A beamline of NSRRC in Taiwan. The XPS measurements were performed under ultra-high vacuum condition (< 10^-6^ bar). The binding energies of collected spectra were calibrated to Au 4*f* _7/2_ of 84 eV for comparison. The quick-XAS measurements were performed in transmission mode at TPS beamline BL-44A in NSRRC. The acquired XANES and EXAFS data were processed with ATHENA, and EXAFS fitting was carried out using the ARTEMIS module of the Demeter software package. Further details are provided in the Supplementary Information.

**Electrochemical measurements.** All the electrochemical performance were carried out in a homemade two-compartment H-cell separated with a Nafion^®^117 membrane at room temperature. The measurements were performed in a conventional three-electrode configuration connected with a CHI 6278e electrochemical workstation (CH Instruments, Inc., USA) with platinum foil as a counter electrode and Hg/HgO as a reference electrode. Working electrodes were prepared by drop-casting the catalyst ink onto carbon cloth. Specifically, the dispersion medium was prepared by mixing 1 mL of 5 wt% Nafion solution with 9 mL of an ethanol/Deionized water (1:1 v/v) mixture. Subsequently, 1 mg of catalyst powder was suspended in 0.6 mL of dispersion medium and sonicated until a homogeneous suspension of catalyst ink was obtained. Finally, 0.03 mL of catalyst ink was drop-cast onto carbon cloth (1 cm^2^), yielding a catalyst loading of 0.05 mg cm^-2^. The potentials reported in this work were all against Reversible Hydrogen Electrode (RHE, E_RHE_) corrected with Equation (1):

$E_{RHE}= E_{Hg/HgO}+0.118+0.059pH$ (1)

LSV measurements were conducted in 1.0 M KOH, with or without 0.1 M KNO_3_, over a potential range of 0.2 to -0.6 V vs. RHE, with 90% *iR*-correction at a scan rate of 5 mV s^-1^. Double layer capacitance (C_dl_) was determined using cyclic voltammograms within the non-Faraday current range (open circuit potential ± 50 mV) under various scan rates of 10-100 mV s^-1^ at 10 mV steps. C_dl_ was calculated using the formula of C_dl_ = *i_v_*/(dE/dt) (where *i_v_* is the difference of i_a_ and i_c_ under OCP; dE/dt represent scan rate). Current density based on electrochemical surface area (*j_ECSA_*) was normalized using the formula of *I*×C_s_/C_dl_ (where *I* is the current based on geometric area; C_s_ represent specific capacitances^1^). Chronoamperometric measurements were performed in 1.0 M KOH with 0.1 M KNO_3_ under various applied potentials for 30 min without *iR*-correction. The EIS was recorded in 1.0 M KOH with 0.1 M KNO_3_ using the AC Impedance mode with a frequency range from 10^6^ to 0.1 Hz under an applied potential of -0.1 V vs. RHE with 10 mV perturbation. All the electrochemical process were performed under Ar purged unless otherwise mentioned.

**Quantitative analysis of NH_3_.** The indophenol method was used to quantify the formation of NH_3_ after each electrolysis. Specifically, the catholyte after the reaction was collected and diluted to a detection range within a calibration curve. Subsequently, 2 mL of diluted electrolyte was mixed with 2 mL of reagent containing salicylic acid (5 wt%) and sodium citrate (5 wt%). Finally, a 1 mL sodium hypochlorite (0.05 M) followed by the addition of 0.2 mL of sodium nitroprusside solution (1 wt%) was further added and thoroughly mixed. After incubating the mixture at room temperature for 2 hours, the UV-vis measurements were carried out using a HITACHI U-3900 spectrophotometer over the wavelength range of 800-500 nm, with the absorbance at 650 nm extracted for NH_3_ quantification. NH_3_ Faradaic efficiency (%) and NH_3_ yield rate (mg_NH3_ mg_cat_^-1^ h^-1^) are calculated with Equation (2) and Equation (3), respectively:

${NH}_{3} Faradaic Efficiency (\%)= \frac{N_{NH3} \times F \times8}{Q}$ (2)

${NH}_{3} Yield rate ({mg}_{NH3} {mg}_{cat.}^{-1} h^{-1})= \frac{N_{NH3}\times17.03 \times{10}^{-3}}{m_{cat.} \times t}$ (3)

Where N_NH3_ is mole of generated NH_3_; F is Faraday constant; 8 is the electron transfer number of NO_3_^-^ convert to NH_3_; Q is the total charge passage during the electrolysis; 17.03 is molecule weight of NH_3_; m_cat._ is the loading amount of catalysts; and t is the electrolysis duration.

**Quantitative analysis of NO_2_^-^.** The Griess test was used to quantify the formation of NO_2_^-^ after each electrolysis. In detail, Griess reagent was prepared with 0.2 mL of H_3_PO_4_ and 1 mL DIW solvent dissolved with 80 mg N-(1-naphthyl) ethylenediamine dihydrochloride and 4 mg sulfanilamide. Additionally, the catholyte after the reaction was collected and diluted to a detection range within a calibration curve. Subsequently, 2 mL of 1 M HCl was added to neutralize the pH, followed by the addition of 0.08 mL of Griess reagent. After incubating the mixture at room temperature for 15 minutes, the UV-vis measurements were carried out over the wavelength range of 650-400 nm, with the absorbance at 540 nm extracted for NO_2_^-^ quantification. NO_2_^-^ Faradaic Efficiency (%) is calculated according to Equation (4):

${NO}_{2}^{-} Faradaic Efficiency (\%)= \frac{N_{NO2-} \times F \times2}{Q}$ (4)

Where N_NO2-_ is mole of generated NO_2_^-^; F is Faraday constant; 2 is the electron transfer number of NO_3_^-^ converted to NO_2_^-^; Q is the total charge passage during the electrolysis.

**Quantitative analysis of NO_3_^-^.** The conversion of NO_3_^-^ was detected under -0.3 V vs. RHE for different durations. To facilitate the evaluation of conversion, the working electrode would be enlarged to 4 cm^2^ while maintaining the identical catalyst loading (0.05 mg cm^-2^). For the NO_3_^-^ detection, the catholyte after the reaction was collected and diluted by a factor of 160. Subsequently, the diluted catholyte was initially neutralized by adding 3 mL of 1 M HCl, followed by adding 0.1 mL of sulfamic acid (0.8%). After incubating the mixture at room temperature for 15 minutes, the UV-vis measurements were carried out over the wavelength range of 300-200 nm, with the absorbance calculated using the formula of A_220_-2A_275_ (where A_220_ and A_275_ is the absorbance at 220 nm and 275 nm, respectively). NO_3_^-^ concentration (mM) is calculated according to Equation (5):

${NO}_{3}^{-} concentration (mM)= \frac{N_{NO3^{-}}}{V}$ (5)

Where N_NO3-_ is mole of generated NO_3_^-^; V is the volume of the catholyte.

**^15^N isotope labeling experiments.** To qualitatively confirm the nitrogen source of the produced NH_3_, the electrolyte consisting of 1.0 M KOH and 0.1 M KNO_3_ was replaced with 1.0 M KOH and 0.1 M K^15^NO_3_. Both electrolytes were subjected to electrolysis at -0.3 V vs. RHE for 0.5 hours. After electrolysis, 580 μL of the electrolyte was mixed with 50 μL of 6 M H_2_SO_4_ and 25 μL of dimethyl sulfoxide-d_6_ (DMSO-d_6_) to obtain the ^1^H NMR spectrum. The ^15^N isotope labeling experiments were conducted using a VARIAN VNMRS-600 NMR spectrometer.

***Operando* quick-scanning XAS measurements.** The Co and Cu K-edge XAS were measured at the TPS beamline BL-44A in NSRRC. *Operando* quick-XAS measurements were performed in the self-assembled Teflon cell. The X-ray beam was transmitted through the Kapton tape and electrolyte and reached the detector for XAS collection in the transmission mode. Platinum was chosen as the counter electrode, while an Hg/HgO electrode served as the reference electrode, with electrolyte of 1.0 M KOH + 0.1 M KNO_3_. For the working electrode preparation, 10 mg of catalyst powder were dissolved in ethanol containing 50 μL of a 5% Nafion solution and sonicated for 20 min to form a homogeneous ink. Subsequently, the mixed solution was uniformly drop-coated onto a 1 x 0.5 cm^2^ carbon cloth and allowed to dry to obtain the working electrode.

***Operando* XES measurements.** The Co and Cu XES measurements were conducted at the beamline BL-12XU of SPring-8 in Japan. *Operando* XES measurements were performed in the self-assembled Teflon cell. The X-ray beam was transmitted through the Kapton tape and electrolyte. In a typical three-electrode setup, the working electrode was a piece of carbon cloth uniformly drop-cast with catalyst ink containing 10 mg of catalyst, with the Hg/HgO electrode and platinum acting as the reference and counter electrodes, respectively, in a solution of 1.0 M KOH + 0.1 M KNO_3_. The fluorescence was separated by the analyzer crystal Si (444) and collected by a silicon drift detector (XR-100CR Si-PIN X-ray detector).

***Operando* Attenuated Total Reflection-Fourier-transform Infrared Absorption Spectroscopy (ATR-FTIR) Measurements**. ATR-FTIR measurements were conducted at the NSRRC on the TLS BL 14A1 beamline. The reflecting surface of a 60° silicon prism (Veemax, 2 cm in diameter) was polished with alumina paste to expose a fresh silicon layer. Then, a solution of 5 mg of CuNCO_3_ catalyst mixed with 100 μL of Nafion ink was dropped onto the fresh surface of the silicon prism after ultrasonic mixing. Subsequently, three small pieces of conductive copper tape were adhered around the circumference of the fresh silicon surface, and finally, an *operando* cell was assembled. The three-electrode cell system used in the experiment included a working electrode with CuNCO_3_ catalyst, a reference electrode (Hg/HgO), and a platinum electrode. This cell was integrated into a Fourier transform infrared spectrometer (Thermo Scientific Nicolet 6700 FT-IR), equipped with a mercury-cadmium telluride (MCT) detector and a specular reflectance accessory set at a 60° angle (Veemax III, Pike Technology). A baseline spectrum was recorded at OCP using a CHI Instrument, followed by a series of applied voltage steps (each overpotential maintained for 3 minutes) within the potential range of 0.1 V to -0.5 V vs. RHE, collecting the corresponding infrared spectra.

**Table S1.** Lattice parameters for CuNCo_3_ were determined by Rietveld refinement of the high-resolution powder XRD data.

| Formula | CuNCo_3_ |
| --- | --- |
| Crystal system | Cubic |
| Space group | Pm-3m |
| a (Å) | 3.74 |
| b (Å) | 3.74 |
| c (Å) | 3.74 |
| α | 90° |
| β | 90° |
| γ | 90° |
| Cell volume (Å^3^) | 52.317 |


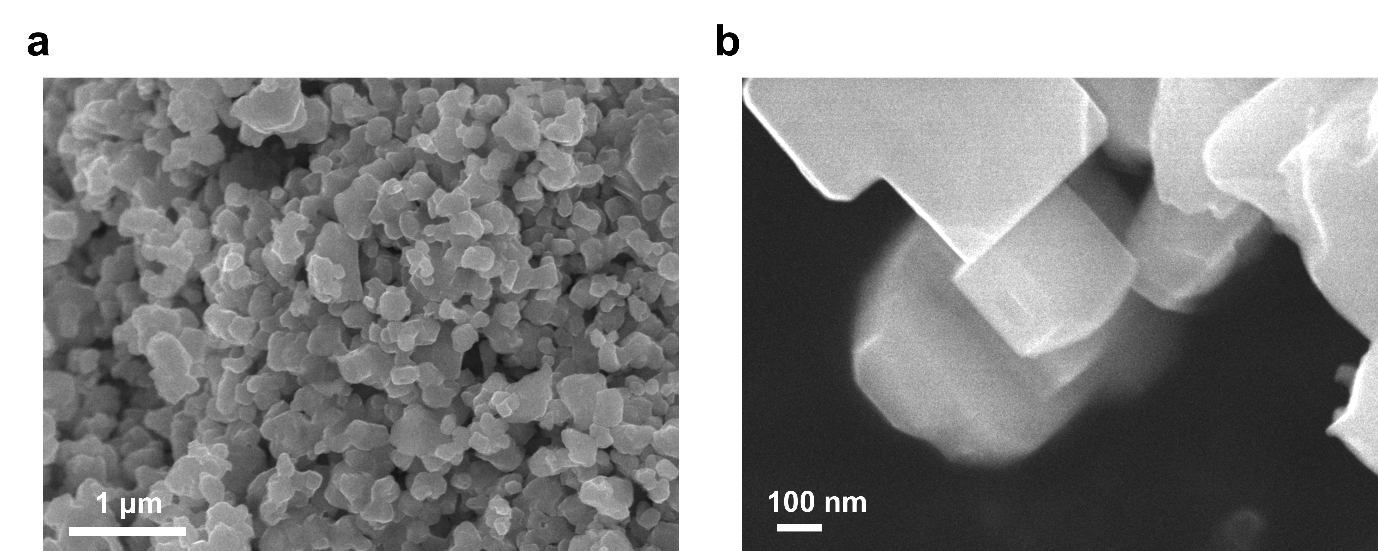


**Figure S1.** **a** SEM image and **b** enlarged SEM image of CuNCo_3_.


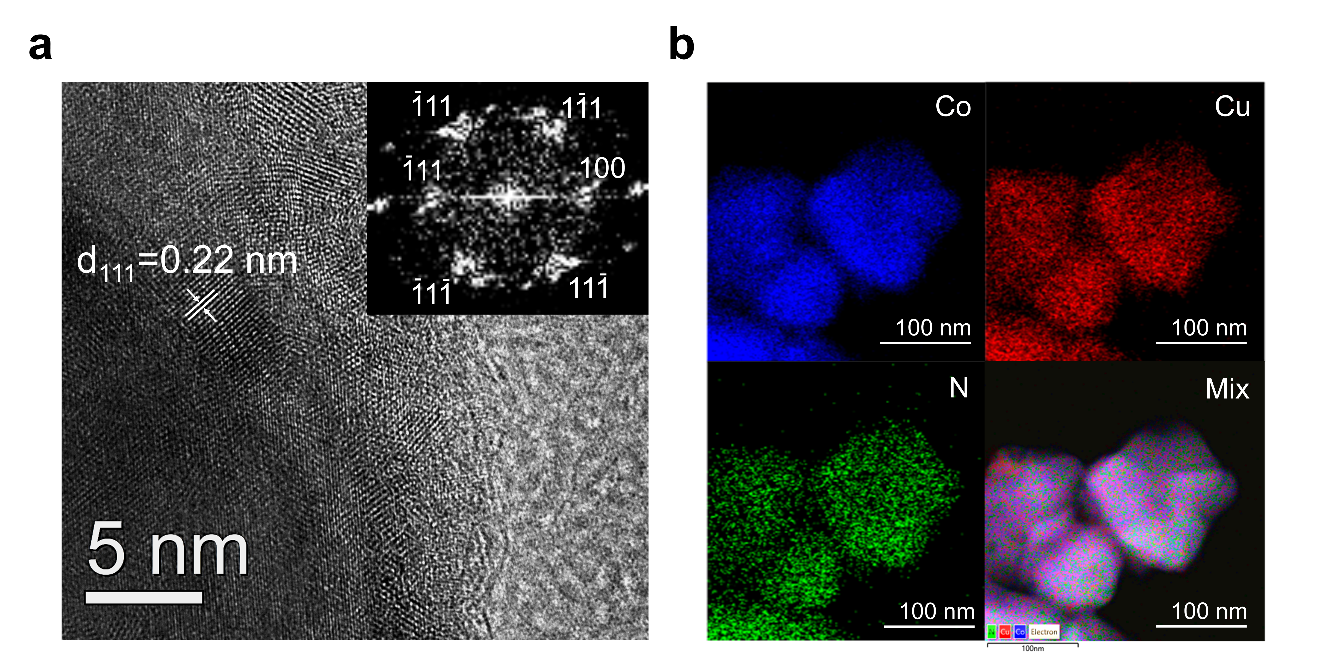


**Figure S2. a** HR-TEM and corresponding FFT pattern of CuNCo_3_ **b** EDS mapping of CuNCo_3_.

**
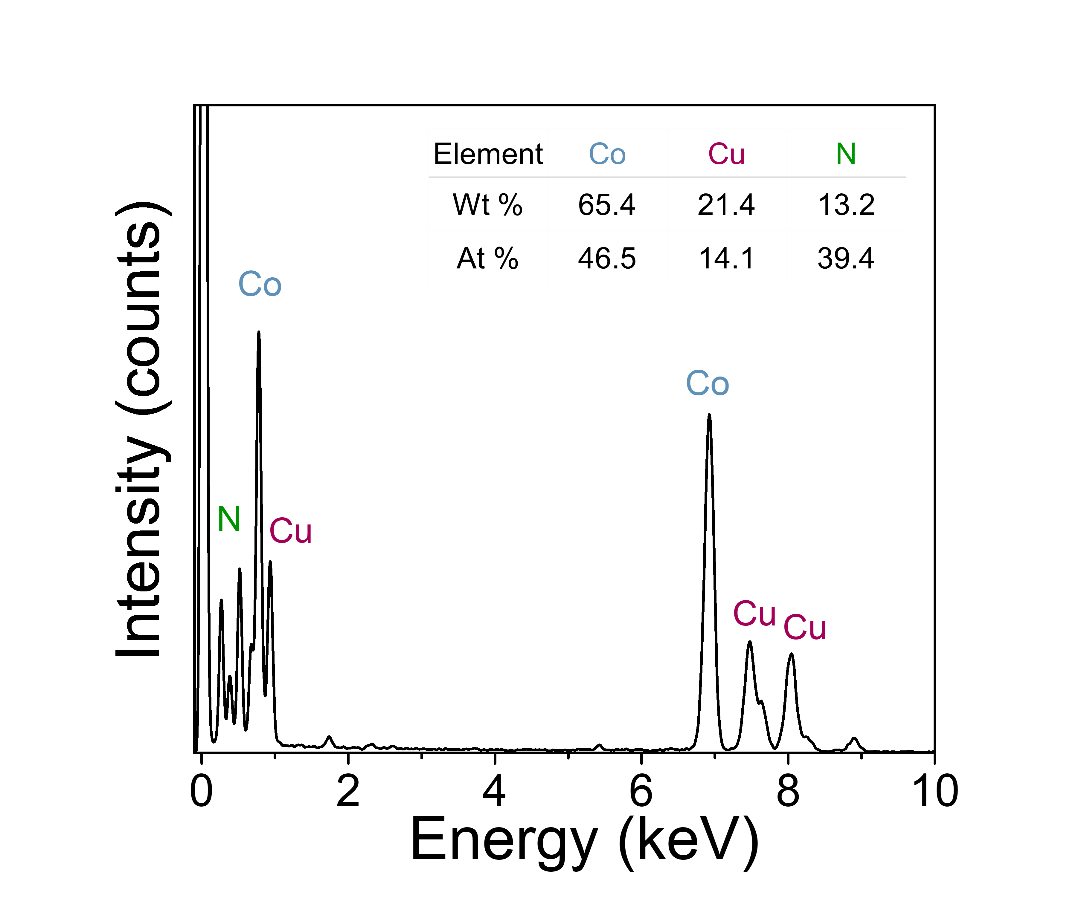
**

**Figure S3.** EDS Composition Ratio of CuNCo_3_ catalyst.

**Table S2.** ICP analysis for CuNCo_3_.

|  | Cu (wt. %) | Co (wt. %) |
| --- | --- | --- |
| CuNCo_3_ | 21.05 | 63.13 |


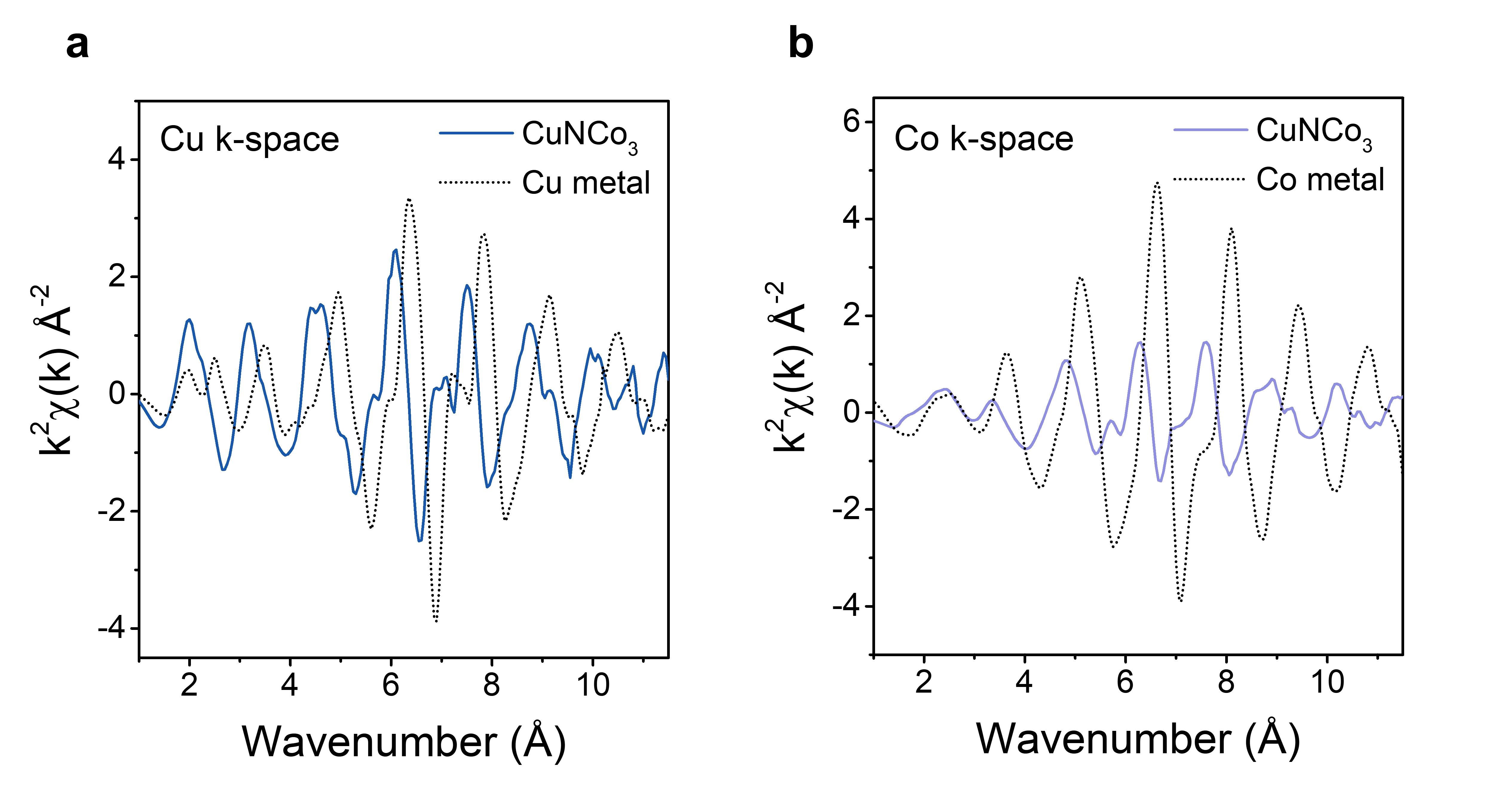


**Figure S4.** Corresponding k-space of **a** Cu K-edge **b** Co K-edge spectra for CuNCo_3_ and metal references.


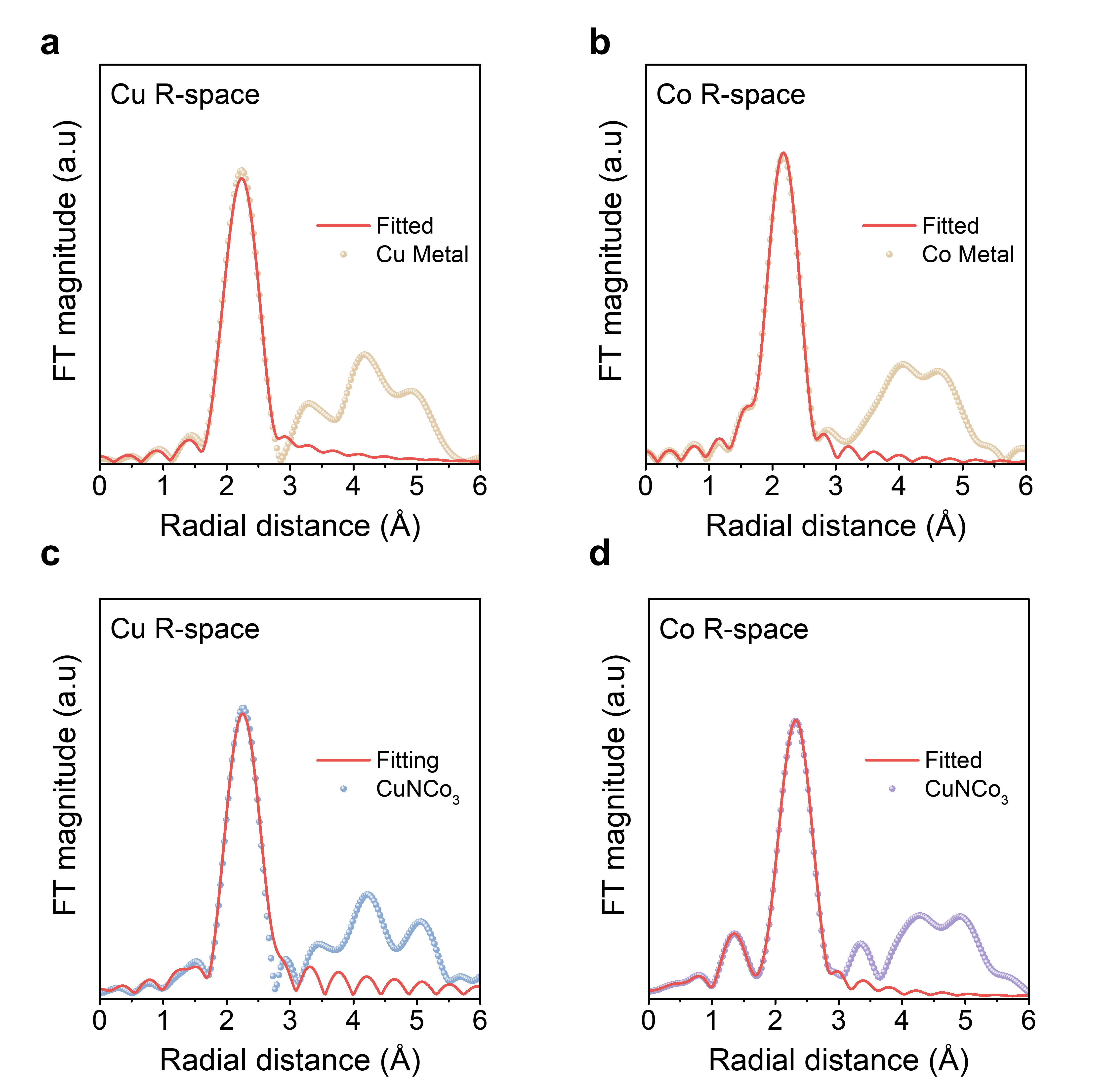


**Figure S5. a,b** Cu and Co EXAFS spectra of metal references. **c,d** Cu and Co EXAFS spectra of CuNCo_3_. The detailed results of the fit are listed in Supplementary Table 3.

**Quantitative EXAFS Curve-Fitting Procedure**

The acquired EXAFS data were processed using the ATHENA module implemented in the Demeter software packages. The data processing involved background subtraction to remove the post-edge signal while preserving the oscillatory features associated with the local atomic structure. Subsequently, normalization to the edge jump was performed to enable meaningful comparison between different samples. The resulting *k*^2^-weighted χ(k) spectra were Fourier-transformed from k-space (3-11 Å^-1^) into R-space using a Hanning window (dk =1 Å^-1^), allowing separation of contributions from different coordination shells for structural analysis.

To obtain quantitative structural parameters for the metal references and CuNCo_3_, a least-squares curve fitting was performed using the ARTEMIS module of the Demeter software packages. The fitting of the multi-path EXAFS data was conducted on the *k*^2^-weighted χ(k) spectra within the R-range of 1 to 3 Å. Key structural parameters, including coordination number (CN), atomic distance (R), Debye-Waller factor (σ^2^), and edge energy shift (ΔE_0_), were treated as free variables during the fitting process. The amplitude reduction factor (S0^2^) was determined from the fitting of metal reference samples, where the CN was fixed at 12, and R, σ^2^, and ΔE_0_ were allowed to vary. The resulting S0^2^ values for Co and Cu were both 0.7. These values were then applied to the Cu and Co components in the CuNCo_3_ sample. During the fitting of CuNCo_3_, the CNs were constrained to positive values to avoid intensity cancellation, while the σ^2^ and ΔE_0_ remained as freely varying parameters. Following this fitting strategy, satisfactory curve-fitting results were obtained, as shown in Supplementary Fig. 5 and 20. The corresponding fitting parameters are summarized in Supplementary Tables 3, 5 and 6.

**Table S3.** Structural parameters extracted from the Cu and Co K-edge EXAFS fitted spectra of CuNCo_3_ and metal reference.

| Element | Sample | Shell | CN | R (Å) | σ^2^(10^-3^Å^-2^) | ΔE_0_(eV) | *R*-factor |
| --- | --- | --- | --- | --- | --- | --- | --- |
| Cu | Cu Metal | Cu-Cu | 12.0±0.6 | 2.54±0.01 | 6.5±0.1 | 4.5±0.6 | 0.008 |
|  | CuNCo_3_ | Cu-Co | 12.0±1.7 | 2.63±0.01 | 8.6±0.1 | 2.8±1.4 | 0.005 |
| Co | Co Metal | Co-Co | 12.0±0.2 | 2.50±0.01 | 6.6±0.1 | 7.5±0.2 | 0.003 |
|  | CuNCo_3_ | Co-N | 2.0±0.2 | 1.85±0.01 | 3.8±0.1 | 8.3±0.2 | 0.001 |
|  |  | Co-Co | 8.0±0.2 | 2.65±0.01 | 9.1±0.1 |  |  |
|  |  | Co-Cu | 4.0±0.2 | 2.60±0.01 | 9.1±0.1 |  |  |


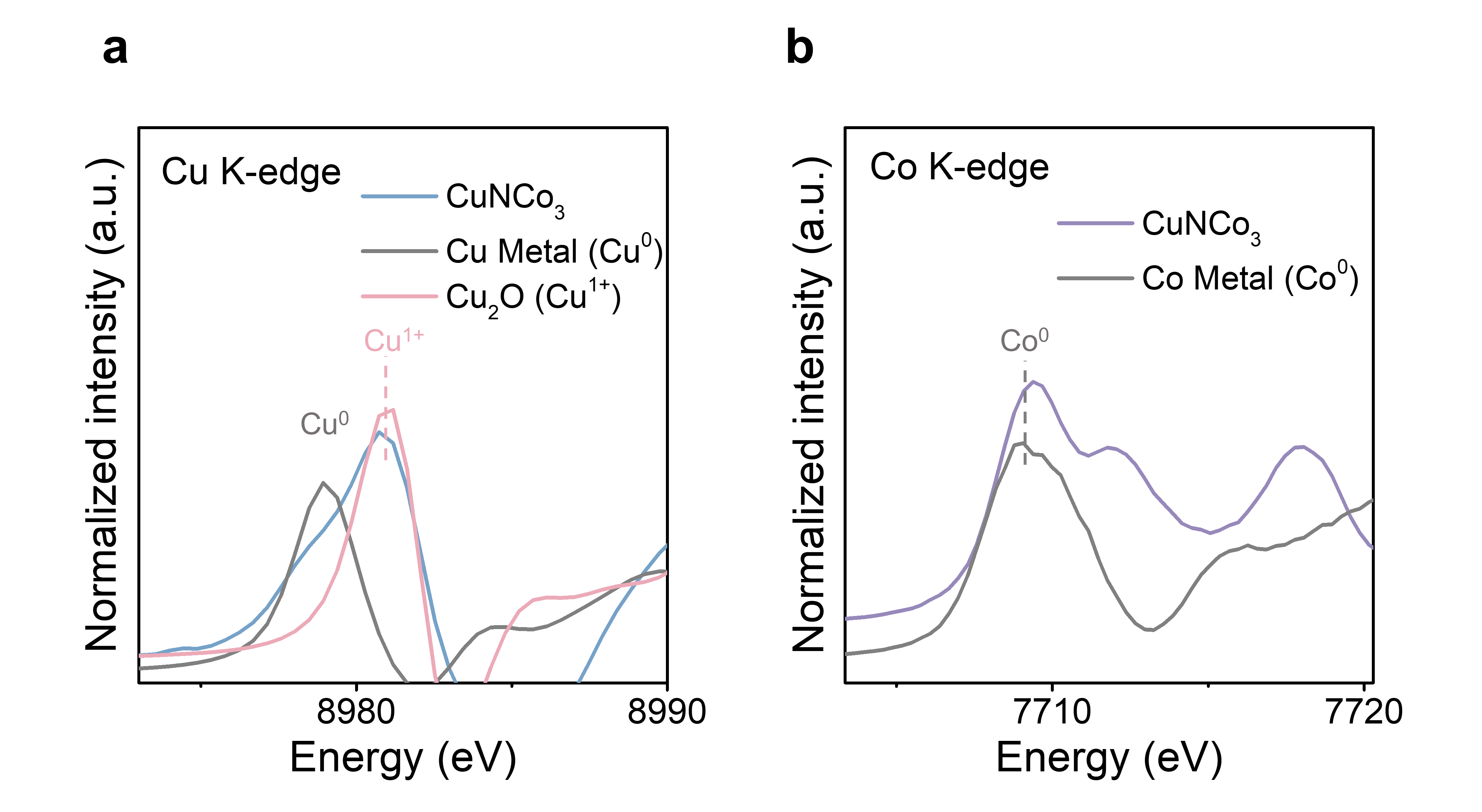


**Figure S6.** Corresponding first-derivative spectra of **a** Cu and **b** Co K-edge for CuNCo_3_.


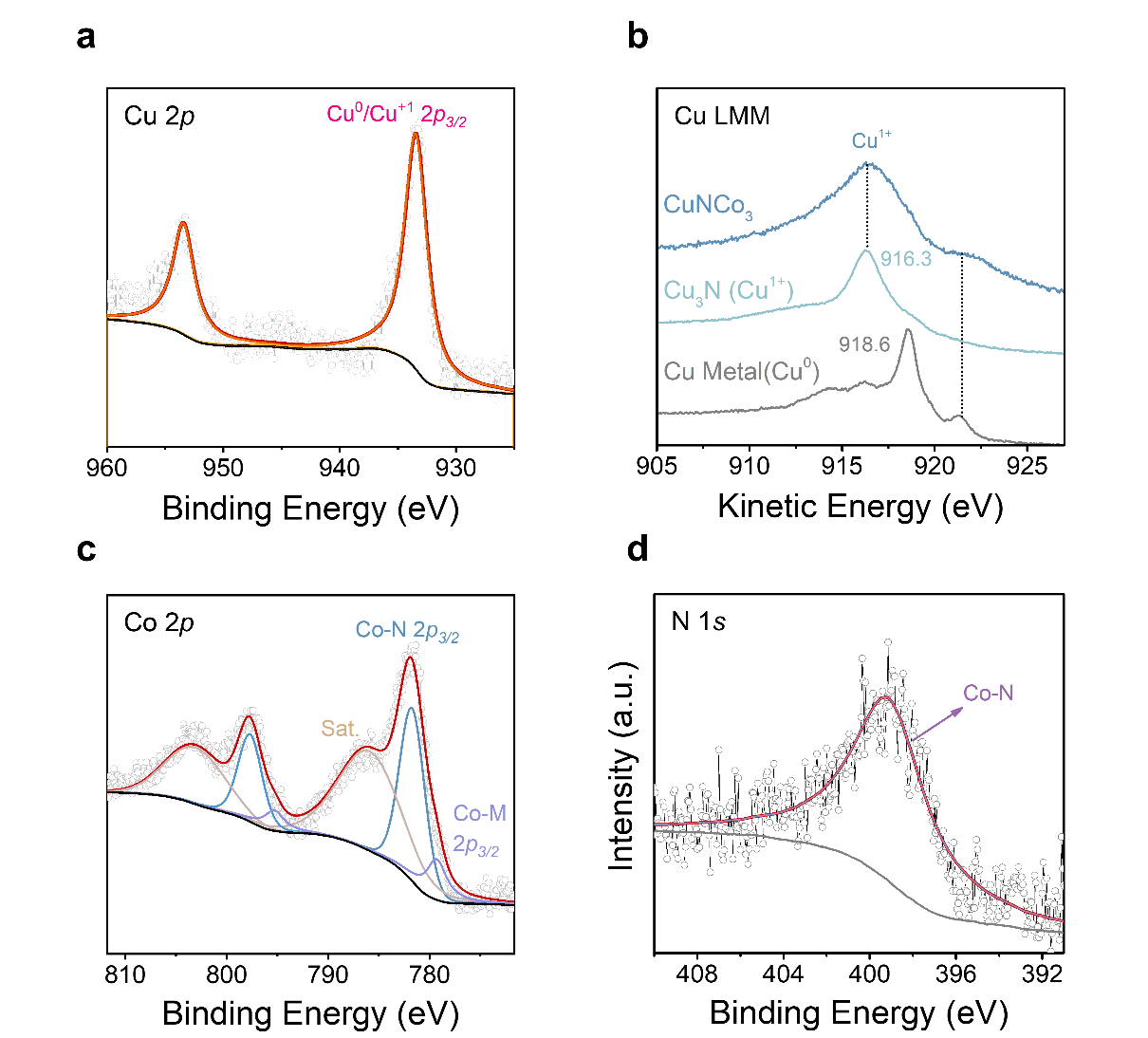


**Figure S7.** XPS of **a** Cu 2*p*, **b** Cu LMM Auger, **c** Co 2*p* and **d** N 1*s* for CuNCo_3_.

**
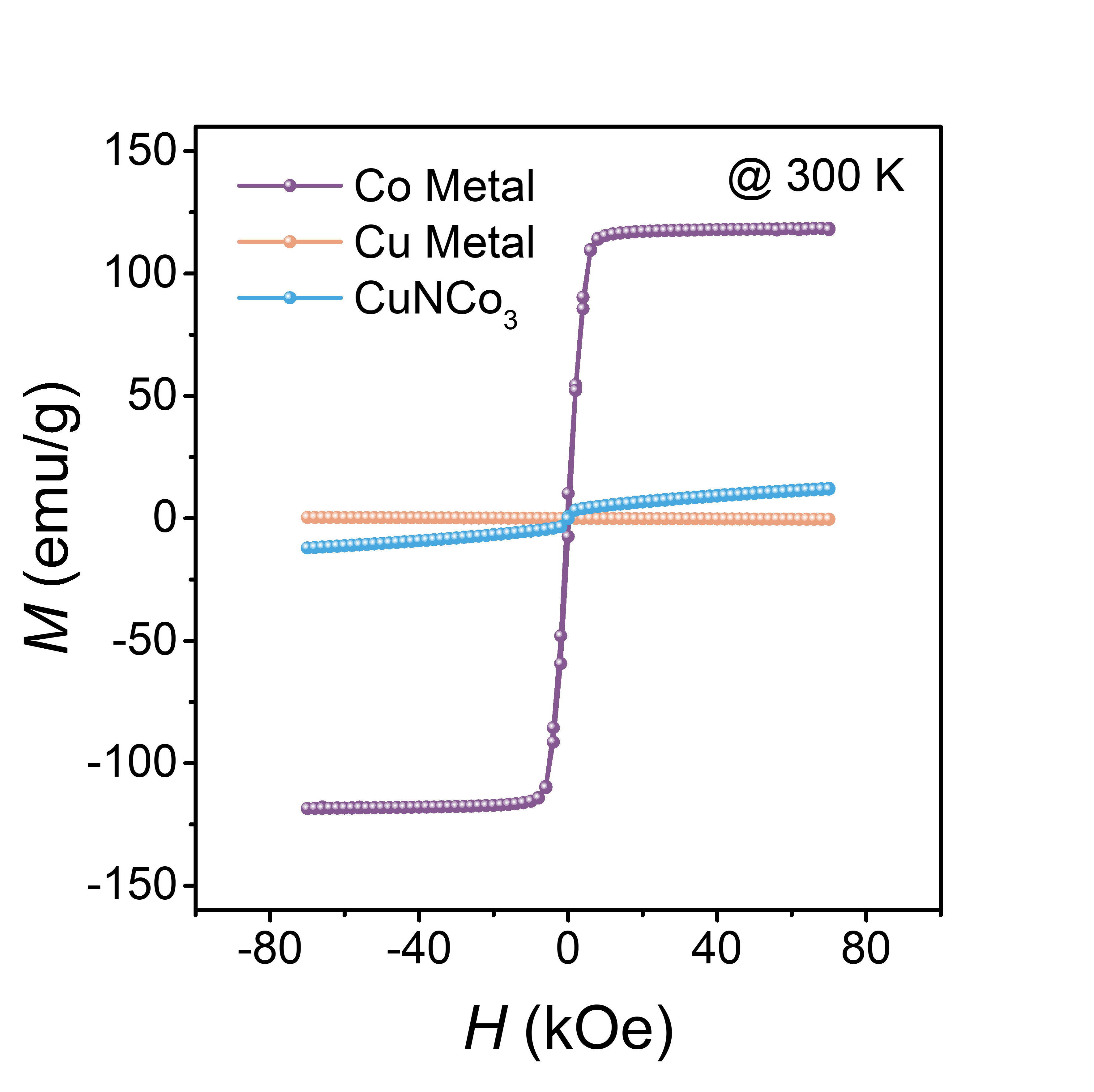
**

**Figure S8.** The magnetic hysteresis loop of CuNCo_3_ compare with metal references at 300 K.


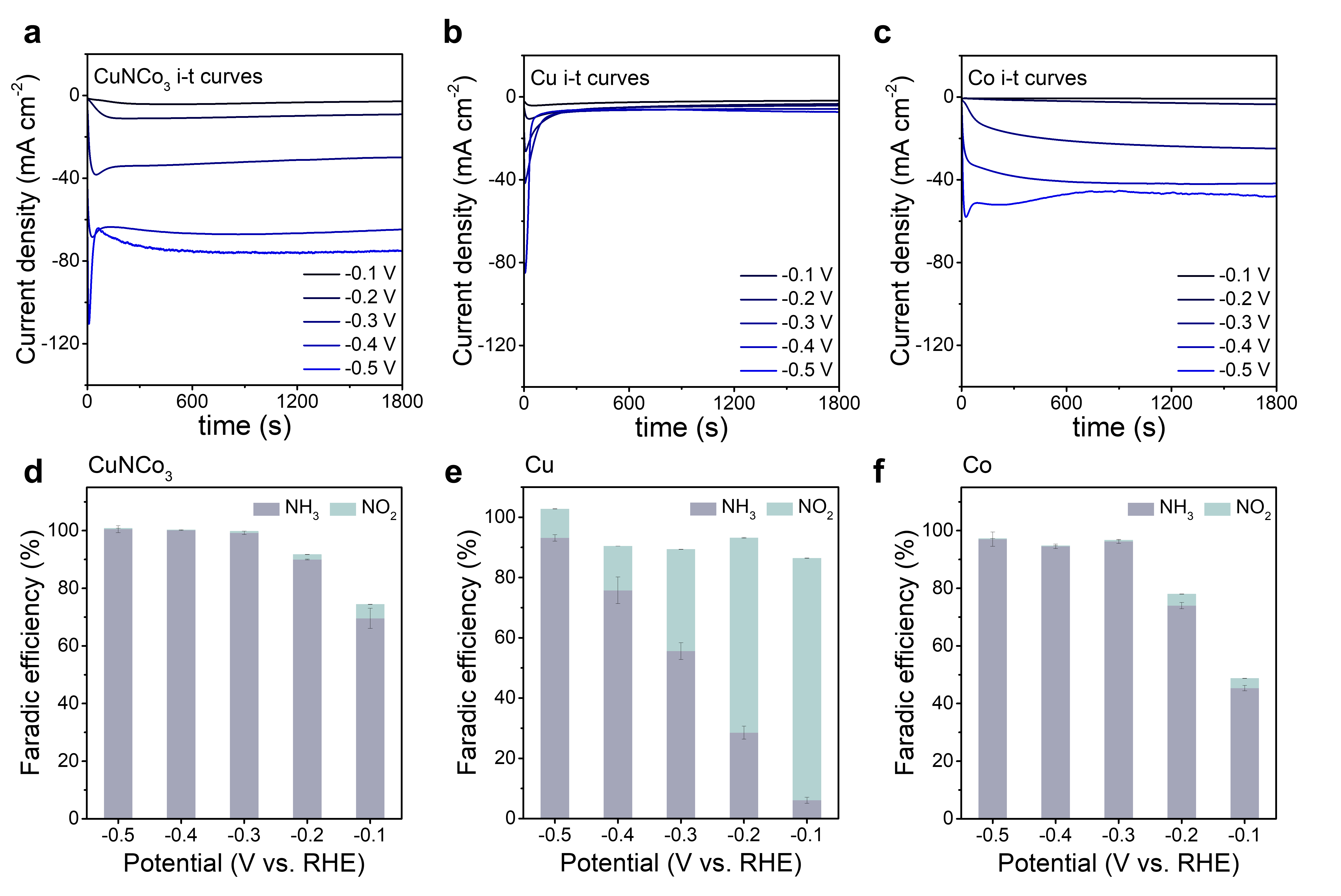


**Figure S9.** Chronopotentiometry tests of **a** CuNCo_3_, **b** Cu and **c** Co catalyst. NH_3_ yield rate of **d** CuNCo_3_, **e** Cu and **f** Co at different potentials.


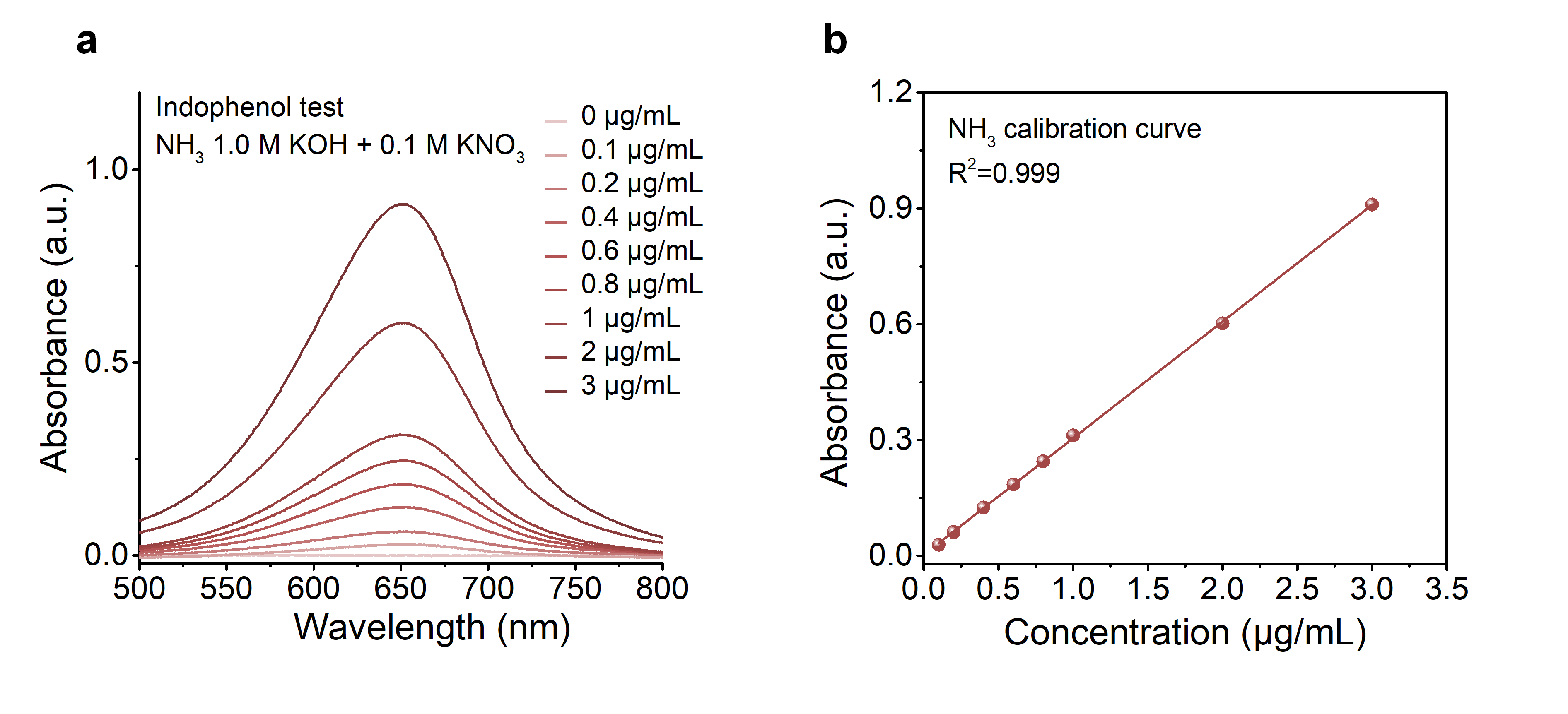


**Figure S10. a** UV-vis Indophenol standard curve for NH_3_. **b** Linear Calibration curve for NH_3_.


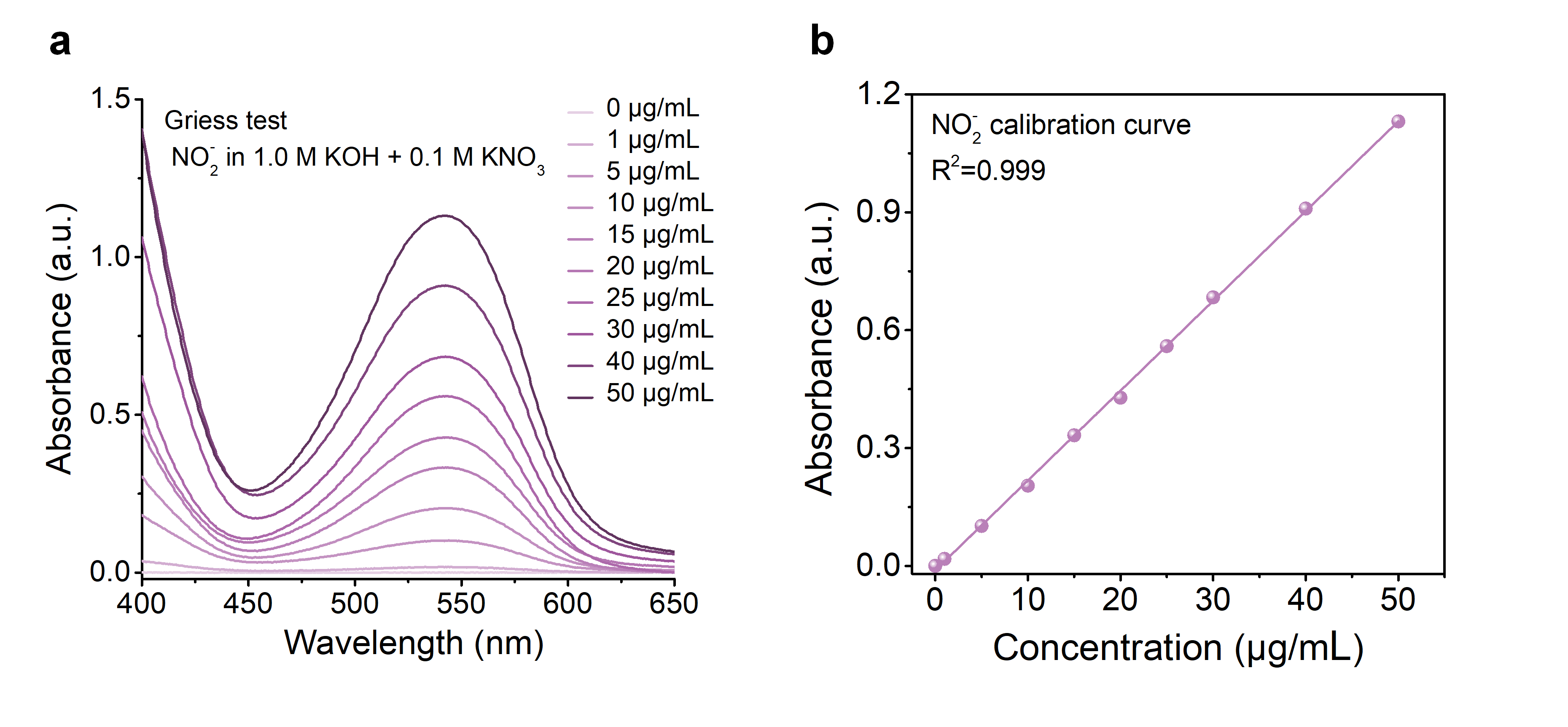


**Figure S11. a** UV-vis Griess standard curve for NO_2_^-^. **b** Linear Calibration curve for NO_2_^-^.


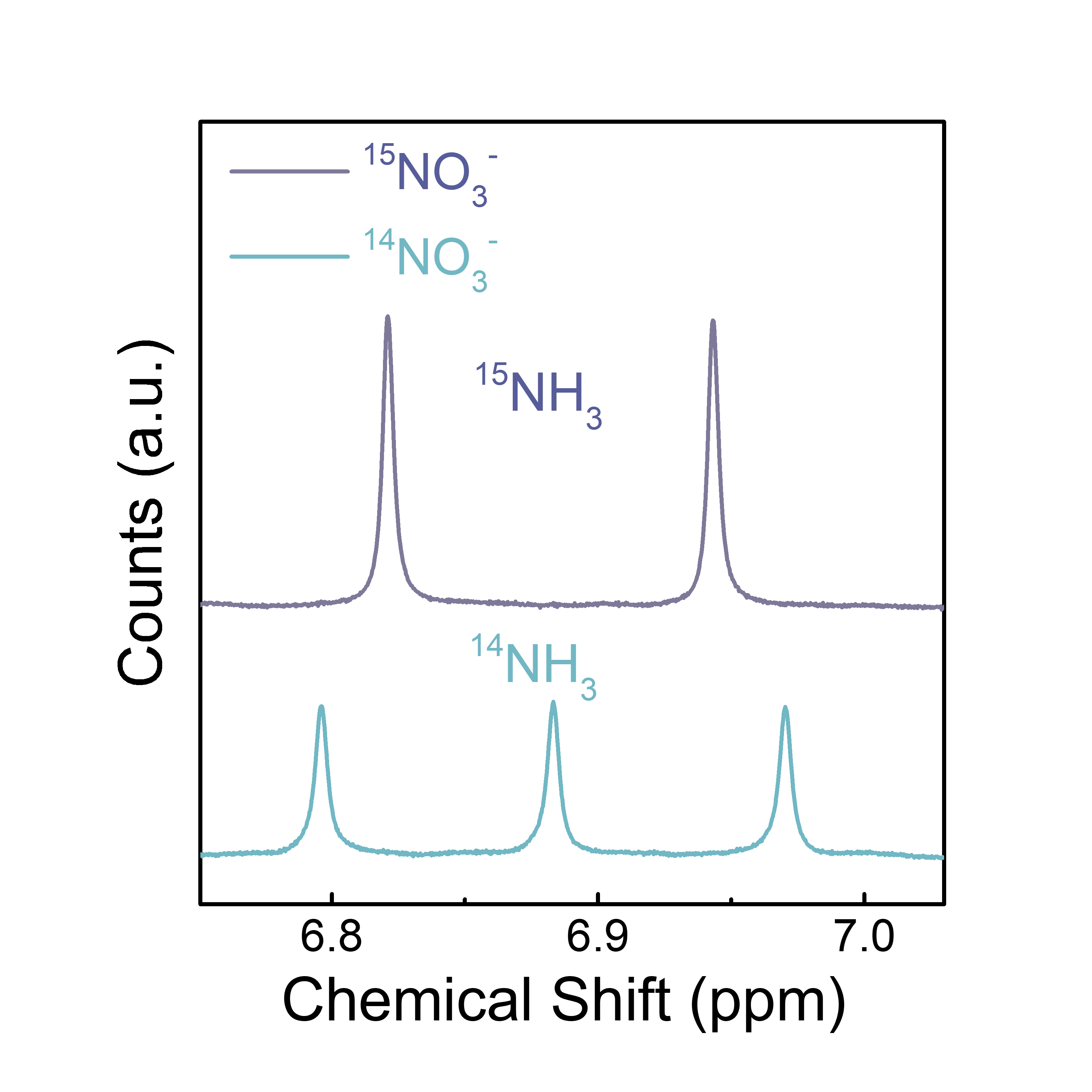


**Figure S12.** ^1^H NMR spectra of ^14^NH_4_^+^ and ^15^NH_4_^+^ formed after 0.5 h of nitrate reduction at -0.3 V vs. RHE, using electrolytes that contained ^14^NO_3_ and ^15^NO_3_ as reactants, respectively.


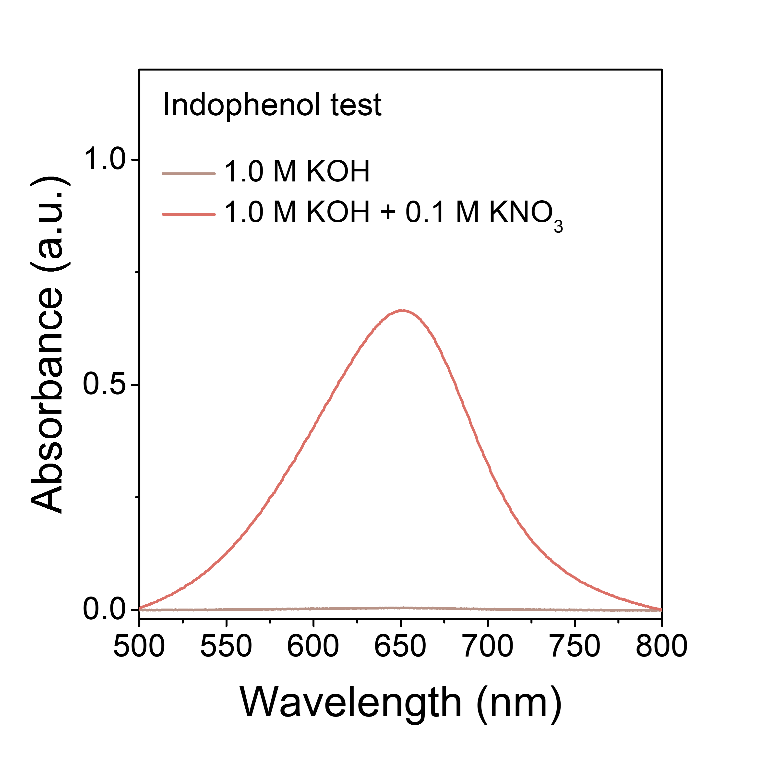


**Figure S13.** Indophenol assays were conducted for CuNCo_3_ in 1.0 M KOH, with and without the addition of 0.1 M KNO_3_, under an applied potential of -0.3 V vs. RHE. After electrolysis, the electrolyte containing 1.0 M KOH and 0.1 M KNO_3_ was diluted 20-fold prior to analysis.


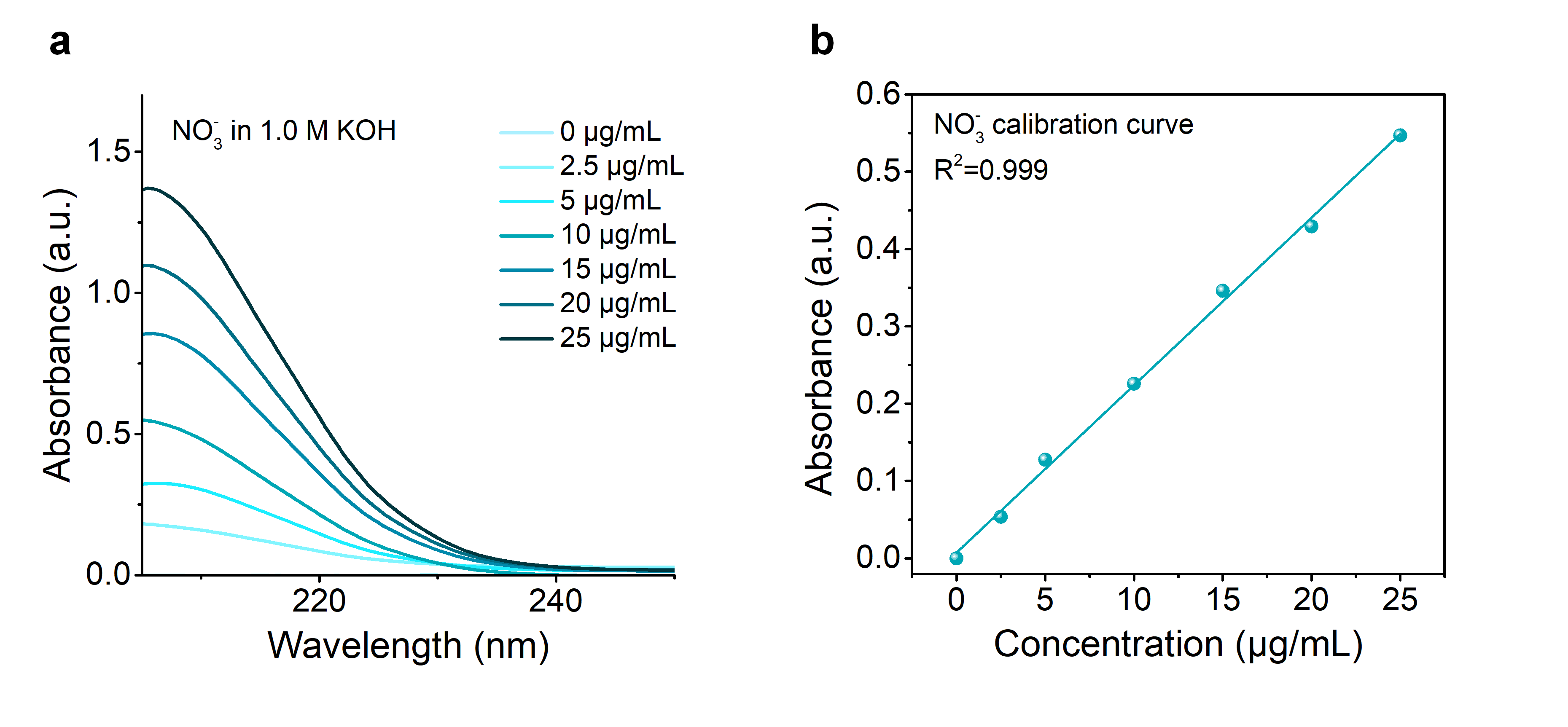


**Figure S14. a** UV-vis standard curve for NO_3_^-^. **b** Linear Calibration curve for NO_3_^-^.


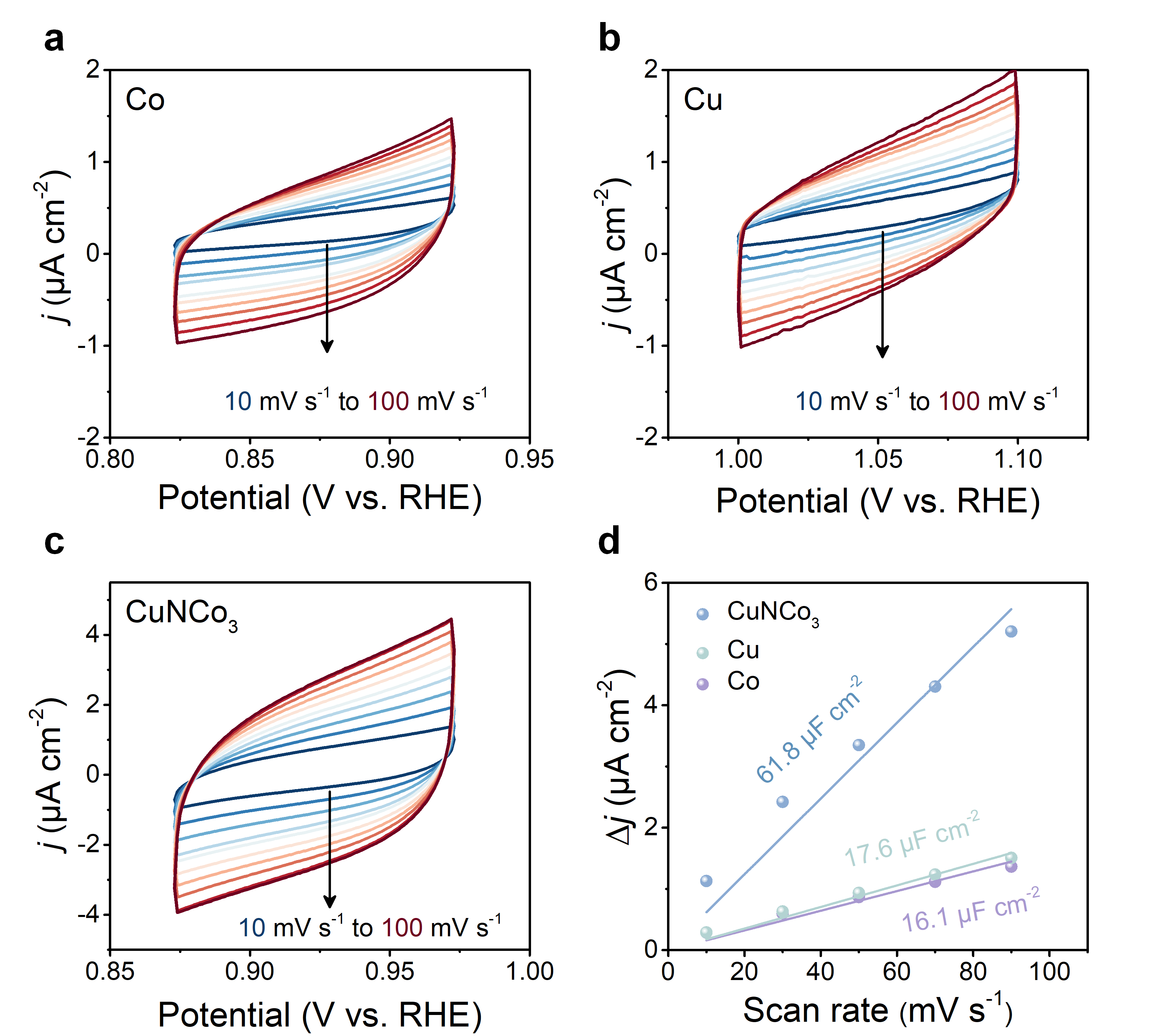


**Figure S15.** CV curves at varied scan rates for **a** Co, **b** Cu and **c** CuNCo_3_. **d** Calculated electrochemical double-layer capacitance.


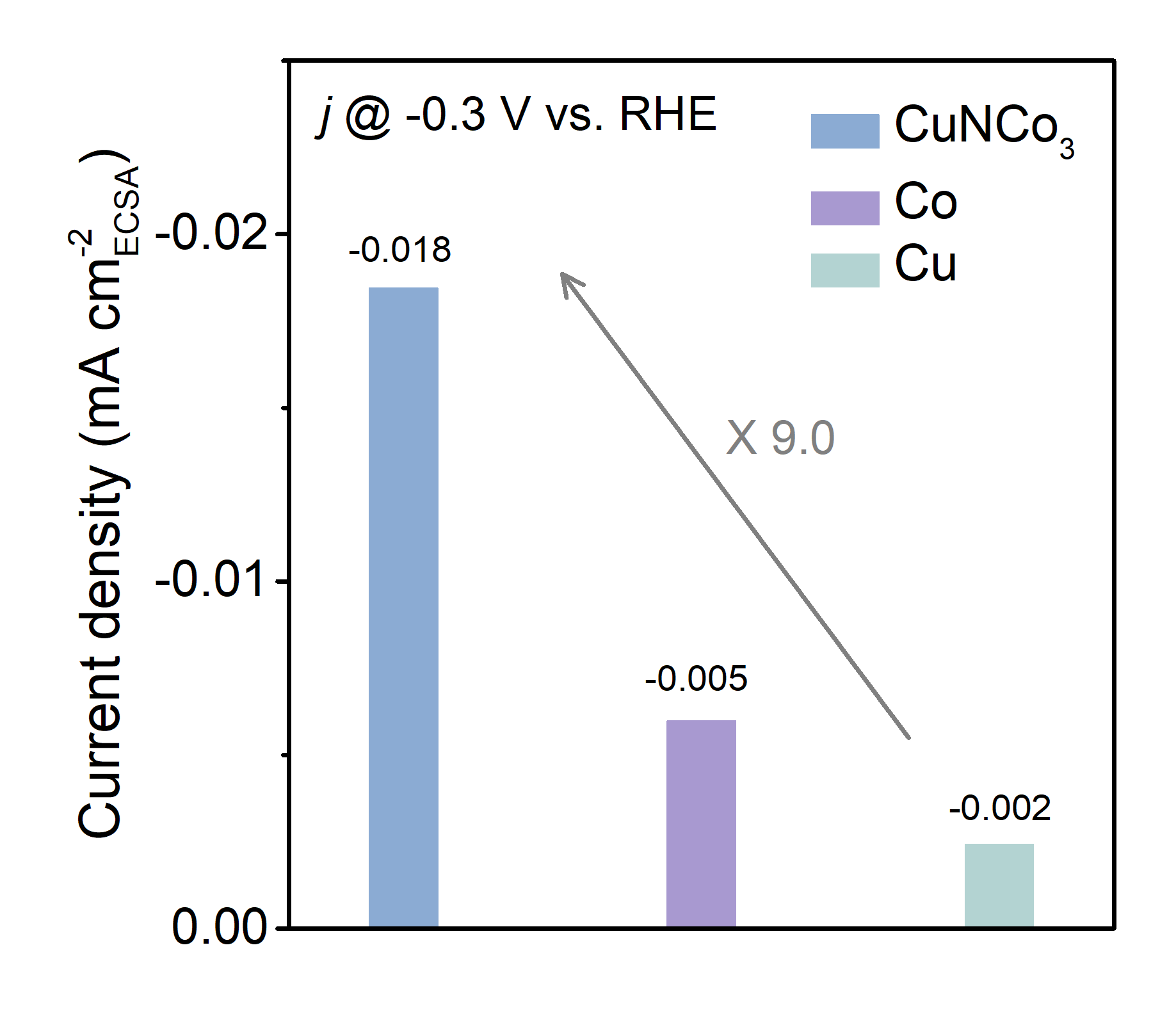


**Figure S16.** ECSA normalized current density recorded at -0.3 V vs. RHE.


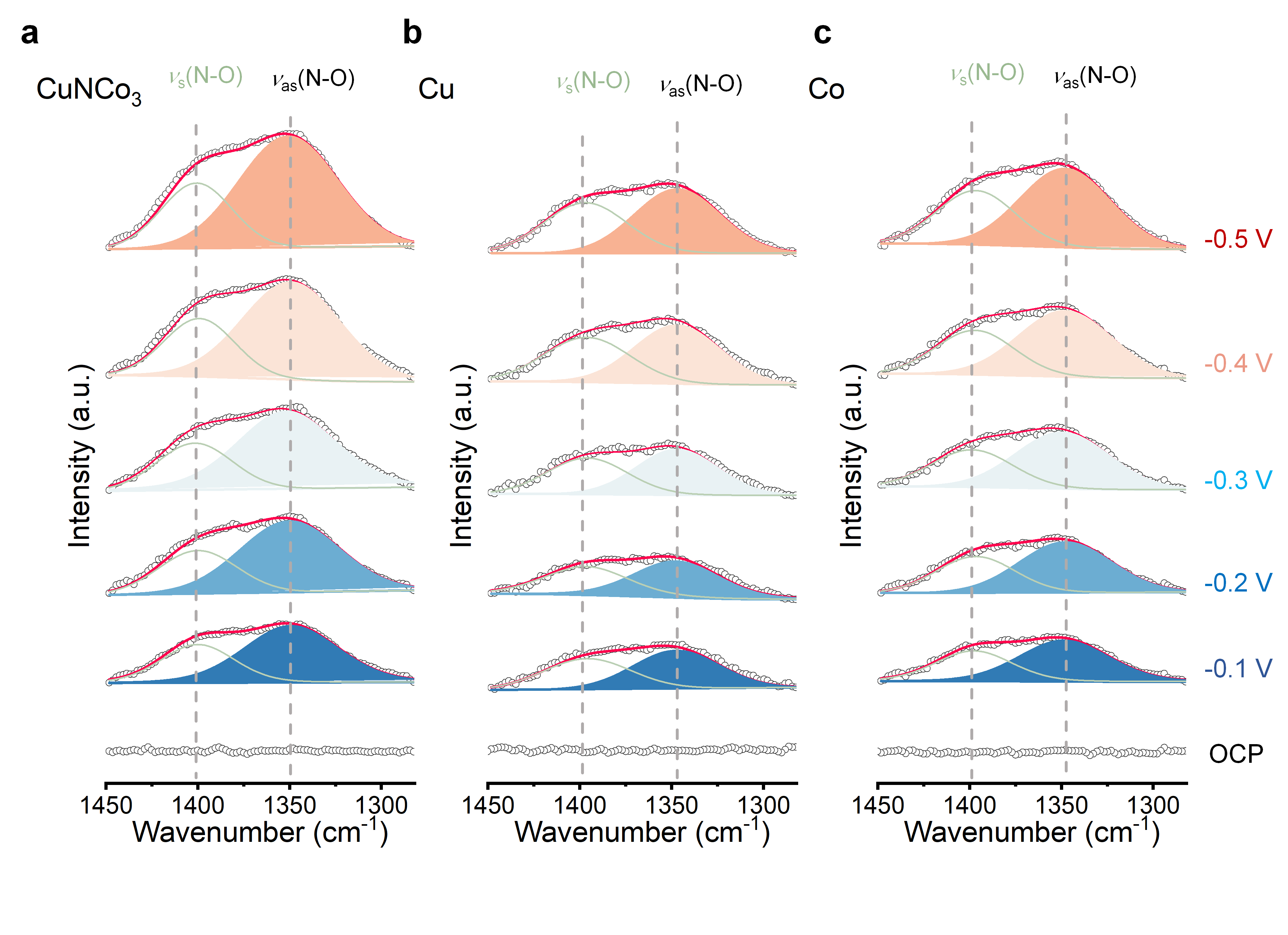


**Figure S17.** *Operando* ATR-FTIR fitted spectra for **a** CuNCo_3_, **b** Cu and **c** Co. The NO_3_^-^ stretching bands are deconvoluted into symmetric and asymmetric peak.


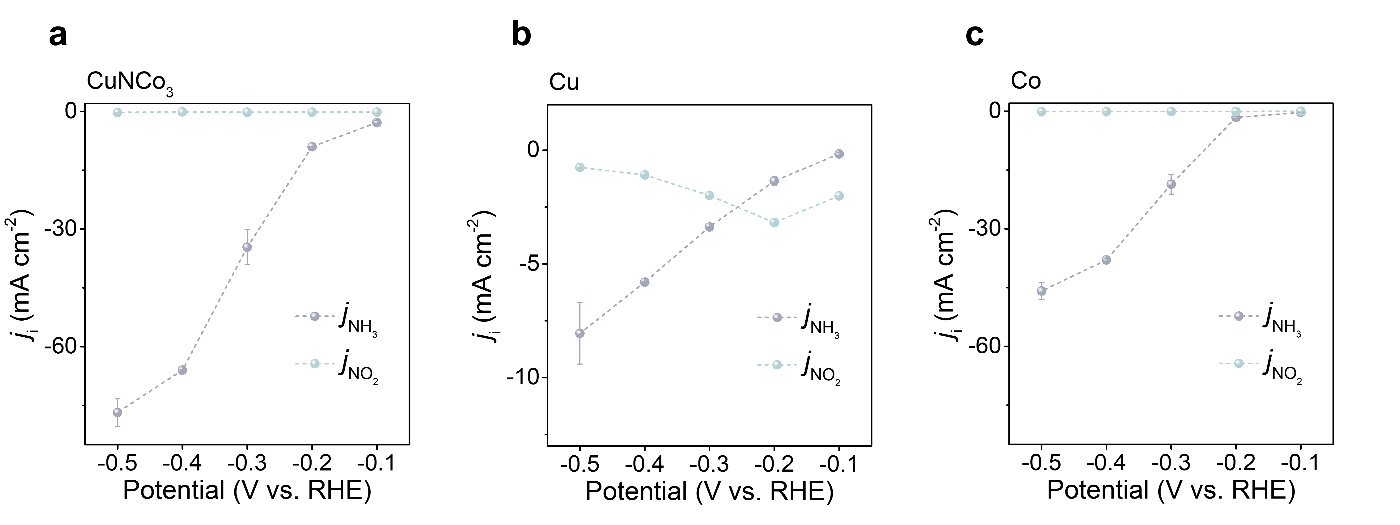


**Figure S18.** Potential‑dependent partial current density of NH_3_ and NO_2_^-^ for **a** CuNCo_3_, **b** Cu and **c** Co.

**Table S4.** Symmetric and asymmetric peak of NO_3_^-^ stretching band areas, extracted from *operando* ATR-FTIR spectra of Cu, Co, and CuNCo_3_ in the range 1300-1450 cm^-1^.

| Sample | CuNCo_3_ | | Cu | | Co | |
| --- | --- | --- | --- | --- | --- | --- |
|  | Area_asym_ | Area_sym_ | Area_asym_ | Area_sym_ | Area_asym_ | Area_sym_ |
| -0.1 V | 0.12 | 0.062 | 0.068 | 0.055 | 0.082 | 0.052 |
| -0.2 V | 0.152 | 0.07 | 0.07 | 0.055 | 0.097 | 0.059 |
| -0.3 V | 0.175 | 0.073 | 0.077 | 0.06 | 0.11 | 0.063 |
| -0.4 V | 0.205 | 0.085 | 0.105 | 0.08 | 0.129 | 0.072 |
| -0.5 V | 0.229 | 0.096 | 0.115 | 0.087 | 0.153 | 0.088 |


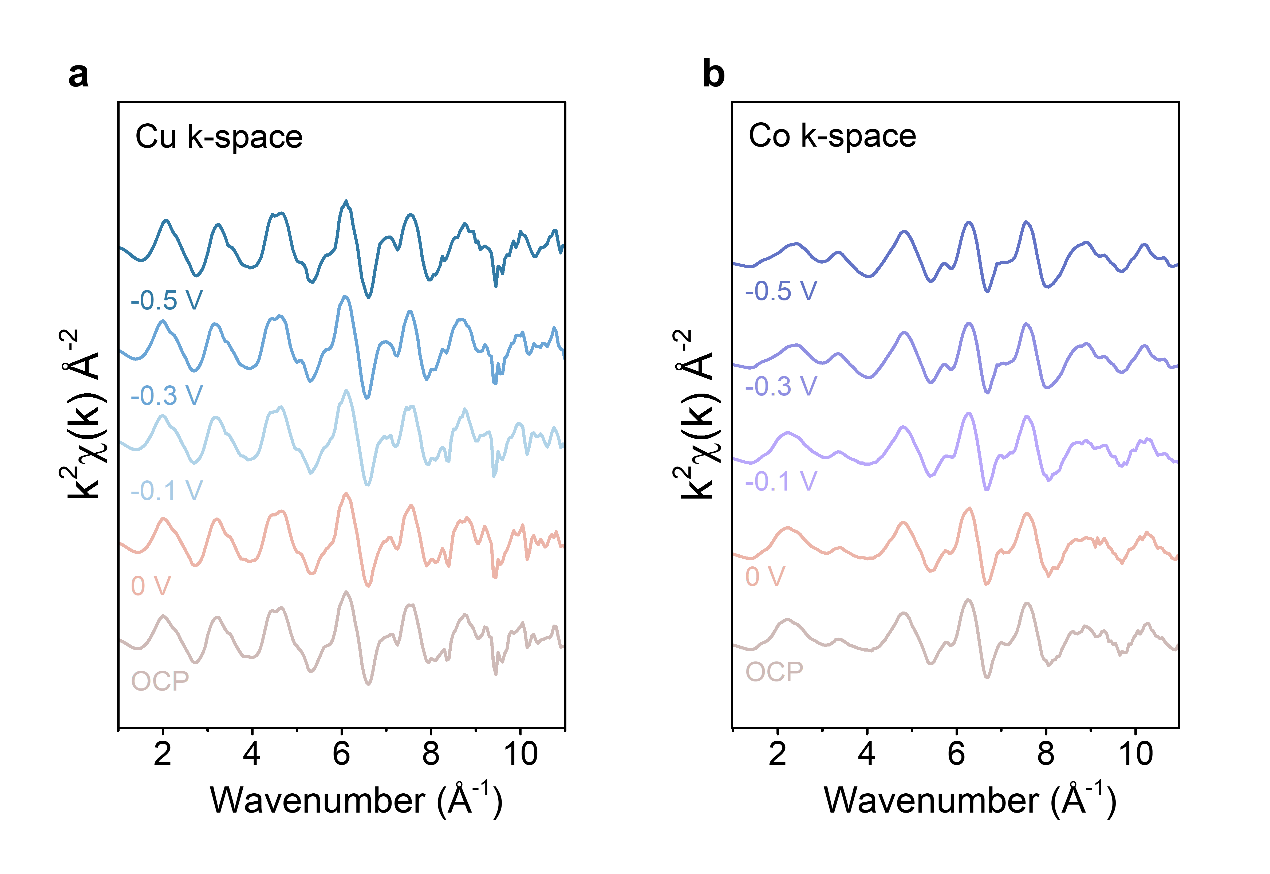


**Figure S19.** Corresponding *operando* k-space **a** Cu **b** Co K-edge EXAFS spectra of CuNCo_3_.


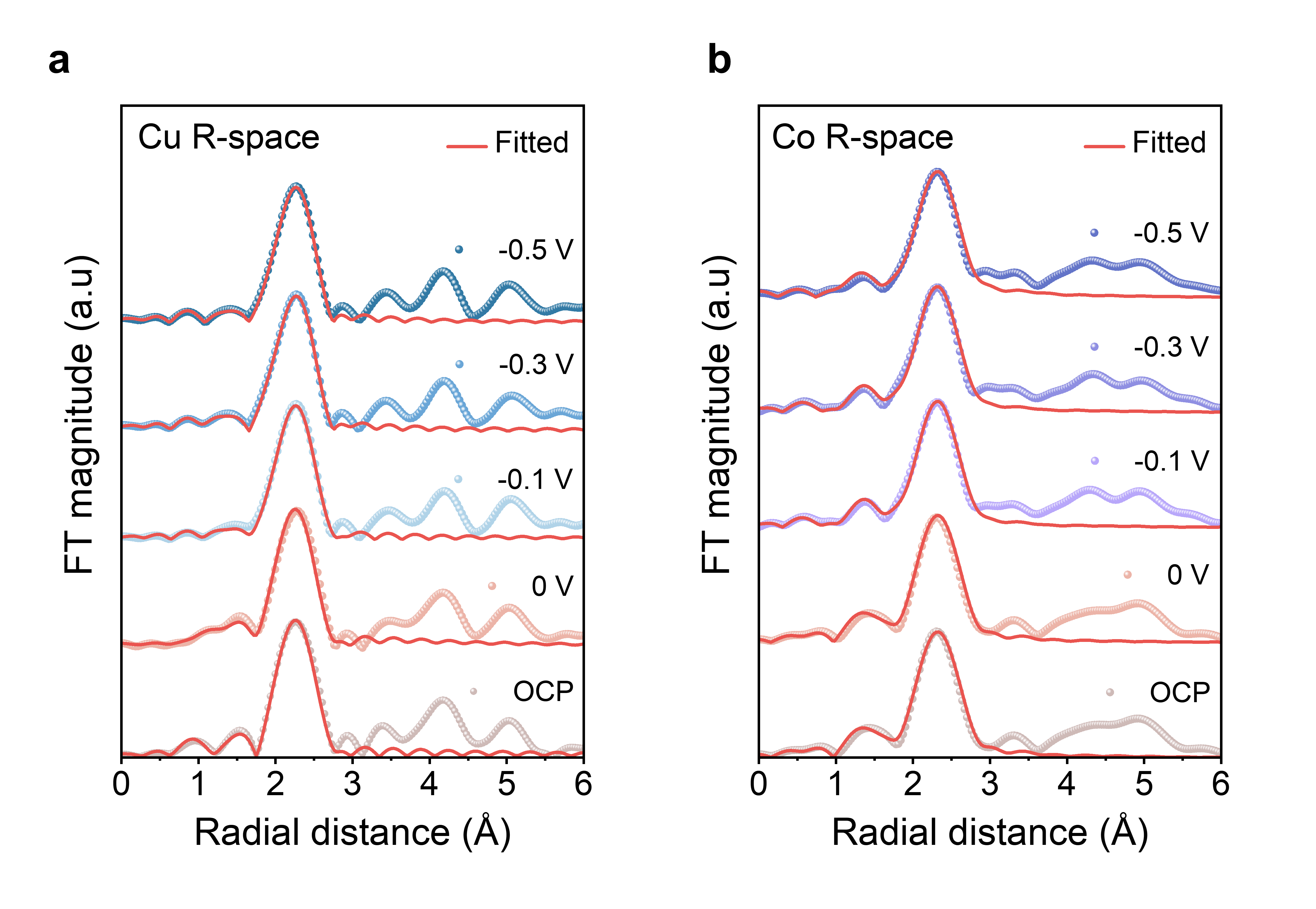


**Figure S20.** *Operando* EXAFS fitted spectra of **a** Cu **b** Co K-edge of CuNCo_3_. The detailed results of the fit are listed in Supplementary Table 5-6.

**Table S5.** Structural parameters extracted from the *Operando* Cu K-edge EXAFS fitted of CuNCo_3_.

| Sample | Shell | CN | R (Å) | σ^2^(10^-3^Å^-2^) | ΔE_0_ (eV) | *R*-factor |
| --- | --- | --- | --- | --- | --- | --- |
| OCP | Cu-Co | 12.0±1.8 | 2.62±0.01 | 9.4±0.1 | 2.1±1.6 | 0.014 |
| 0 V | Cu-Co | 12.0±1.5 | 2.63±0.01 | 9.6±0.1 | 2.8±1.4 | 0.008 |
| -0.1 V | Cu-Co | 12.0±1.6 | 2.61±0.01 | 9.7±0.1 | 1.5±1.4 | 0.013 |
| -0.3 V | Cu-Co | 12.0±1.6 | 2.61±0.01 | 9.6±0.1 | 1.0±1.4 | 0.011 |
| -0.5 V | Cu-Co | 12.0±1.6 | 2.61±0.01 | 9.6±0.1 | 0.7±1.4 | 0.009 |

| **Table S6.** Structural parameters extracted from the *Operando* Co K-edge EXAFS fitted of CuNCo_3_. | | | | | | |
| --- | --- | --- | --- | --- | --- | --- |
| Sample | Shell | CN | R (Å) | σ^2^(10^-3^Å^-2^) | ΔE_0_ (eV) | *R*-factor |
| OCP | Co-N | 2.0±0.4 | 1.87±0.02 | 6.0±0.1 | 9.3±0.6 | 0.013 |
|  | Co-Co | 8.0±0.5 | 2.68±0.01 | 9.8±0.1 |  |  |
|  | Co-Cu | 4.0±0.5 | 2.58±0.01 | 9.8±0.1 |  |  |
| 0 V | Co-N | 2.0±0.4 | 1.88±0.02 | 6.0±0.1 | 9.3±0.6 | 0.013 |
|  | Co-Co | 8.0±0.5 | 2.68±0.01 | 9.7±0.1 |  |  |
|  | Co-Cu | 4.0±0.5 | 2.58±0.01 | 9.7±0.1 |  |  |
| -0.1 V | Co-N | 1.9±0.4 | 1.87±0.02 | 7.2±0.1 | 8.6±0.6 | 0.017 |
|  | Co-Co | 8.0±0.6 | 2.68±0.01 | 9.9±0.1 |  |  |
|  | Co-Cu | 4.0±0.6 | 2.58±0.01 | 9.9±0.1 |  |  |
| -0.3 V | Co-N | 1.9±0.4 | 1.86±0.02 | 7.3±0.1 | 8.3±0.6 | 0.018 |
|  | Co-Co | 8.0±0.6 | 2.68±0.01 | 9.9±0.1 |  |  |
|  | Co-Cu | 4.0±0.6 | 2.57±0.01 | 9.9±0.1 |  |  |
| -0.5 V | Co-N | 1.8±0.4 | 1.86±0.02 | 7.8±0.1 | 8.6±0.5 | 0.01 |
|  | Co-Co | 8.0±0.4 | 2.67±0.01 | 9.8±0.1 |  |  |
|  | Co-Cu | 4.0±0.4 | 2.57±0.01 | 9.8±0.1 |  |  |


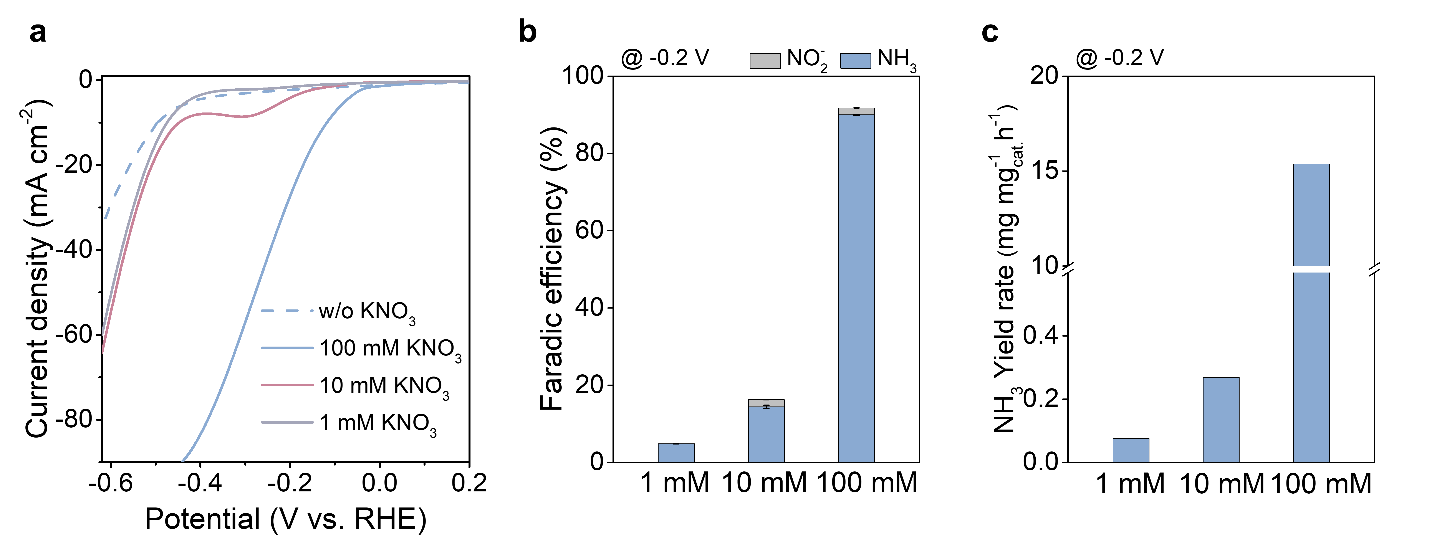


**Figure S21 a** LSV curves of CuNCo_3_ measured in 1.0 M KOH with different NO_3_^-^ concentrations. Corresponding **b** NH_3_ Faradaic efficiency and **c** yield rate at -0.2 V vs. RHE.

**
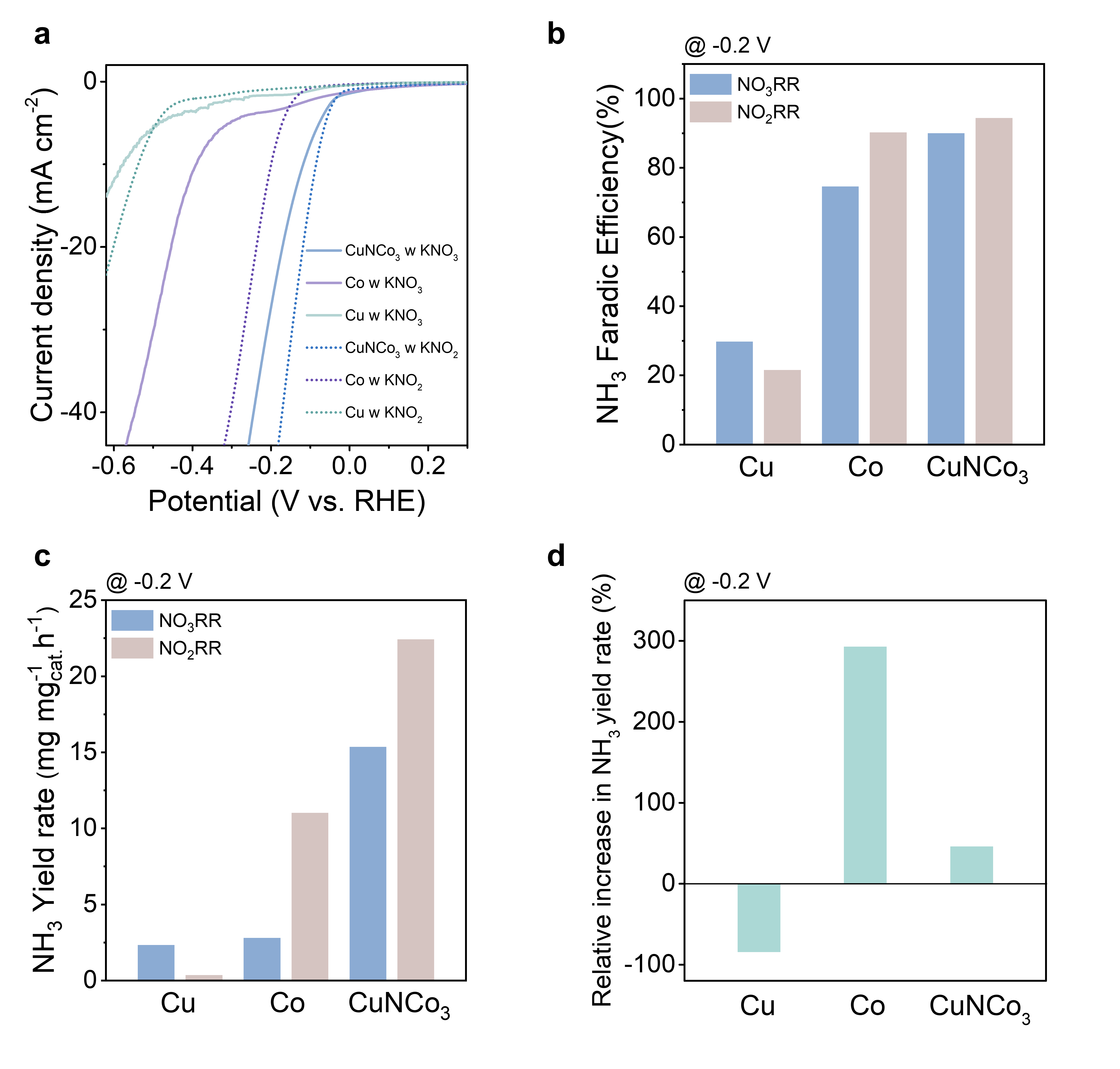
**

**Figure S22 a** LSV curves of Cu, Co, and CuNCo_3_ for NO_3_RR in 0.1 M KNO_3_ + 1 M KOH and for NO_2_RR in 0.1 M KNO_2_ + 1 M KOH. **b** NH_3_ FE of each catalyst under NO_2_RR at -0.2 V vs. RHE. **c** Corresponding NH_3_ yield rate under NO_2_RR. **d** Relative increase in NH_3_ yield rate in NO_2_RR compared with NO_3_RR for Cu, Co, and CuNCo_3_, calculated by NH_3_ yield rate (NO_2_RR-NO_3_RR)/NO_3_RR (%).

**
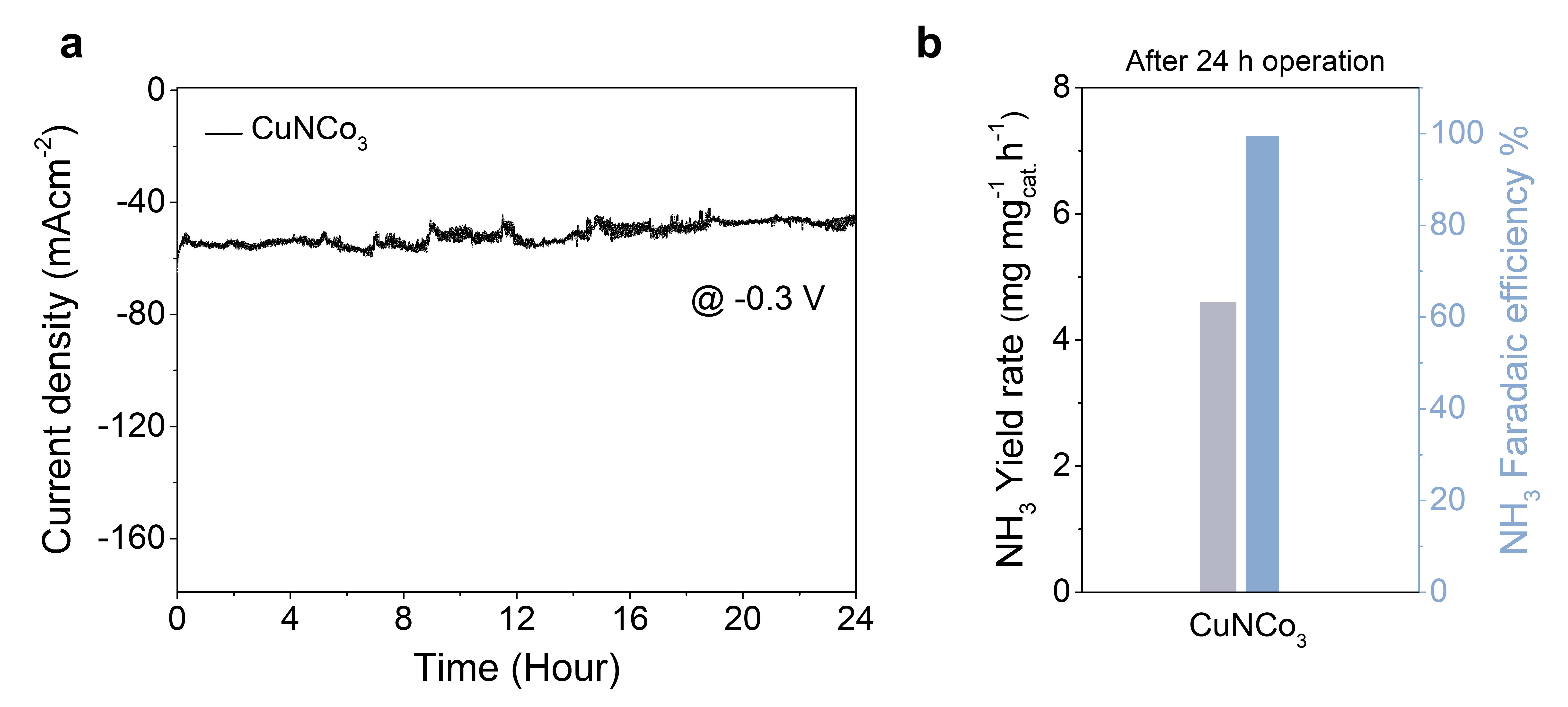
**

**Figure S23. a** chronoamperometry stability test over 24 h of operation at -0.3 V vs. RHE **b** corresponding NH_3_ yield rate and Faradaic efficiency over 24 h of operation.


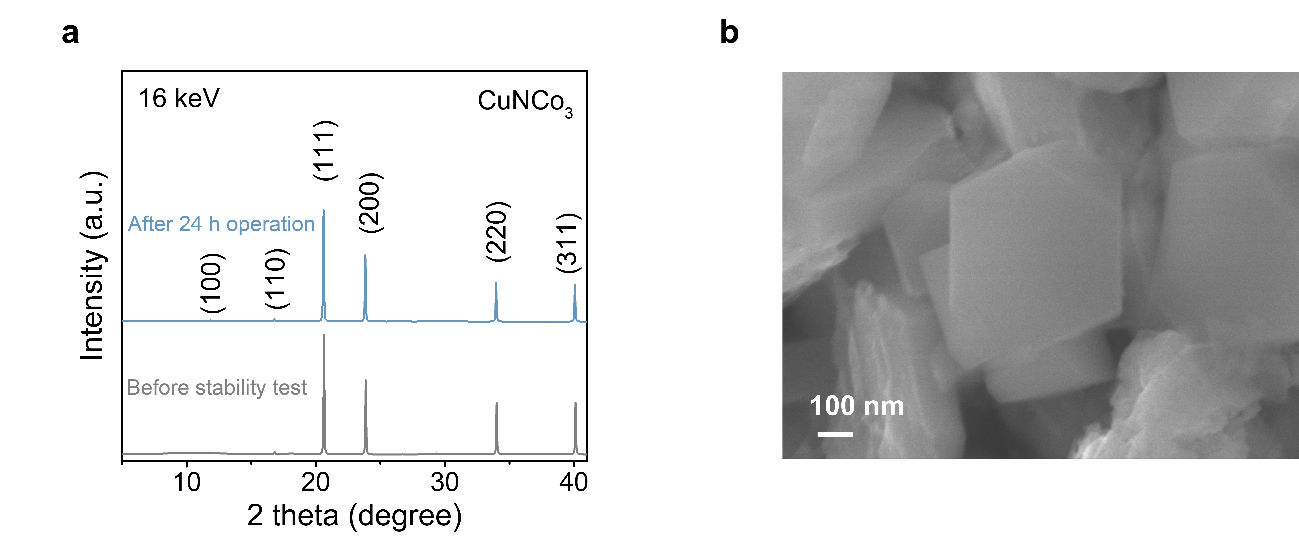


**Figure S24. a** XRD patterns of CuNCo_3_ collected before and after 24 h of operation at -0.3 V vs. RHE. **b** SEM image of CuNCo_3_ after 24 h of operation at -0.3 V vs. RHE.

**Table S7.** ICP analysis of dissolved Cu and Co after 24 h operation for CuNCo_3_.

|  | Cu | Co |
| --- | --- | --- |
| CuNCo_3_ | < 10 ppb | < 10 ppb |

**References**

1. McCrory, C. C., et al. Benchmarking heterogeneous electrocatalysts for the oxygen evolution reaction. *J. Am. Chem. Soc.* **135,** 16977-16987 (2013).
